# invMap: a sensitive mapping tool for long noisy reads with inversion structural variants

**DOI:** 10.1093/bioinformatics/btad726

**Published:** 2023-12-07

**Authors:** Ze-Gang Wei, Peng-Yu Bu, Xiao-Dan Zhang, Fei Liu, Yu Qian, Fang-Xiang Wu

**Affiliations:** School of Physics and Optoelectronics Technology, Baoji University of Arts and Sciences, Baoji 721016, China; Division of Biomedical Engineering, Department of Computer Science and Department of Mechanical Engineering, University of Saskatchewan, Saskatoon, SK S7N 5A9, Canada; School of Physics and Optoelectronics Technology, Baoji University of Arts and Sciences, Baoji 721016, China; School of Physics and Optoelectronics Technology, Baoji University of Arts and Sciences, Baoji 721016, China; School of Physics and Optoelectronics Technology, Baoji University of Arts and Sciences, Baoji 721016, China; School of Physics and Optoelectronics Technology, Baoji University of Arts and Sciences, Baoji 721016, China; Division of Biomedical Engineering, Department of Computer Science and Department of Mechanical Engineering, University of Saskatchewan, Saskatoon, SK S7N 5A9, Canada

## Abstract

**Motivation:**

Longer reads produced by PacBio or Oxford Nanopore sequencers could more frequently span the breakpoints of structural variations (SVs) than shorter reads. Therefore, existing long-read mapping methods often generate wrong alignments and variant calls. Compared to deletions and insertions, inversion events are more difficult to be detected since the anchors in inversion regions are nonlinear to those in SV-free regions. To address this issue, this study presents a novel long-read mapping algorithm (named as invMap).

**Results:**

For each long noisy read, invMap first locates the aligned region with a specifically designed scoring method for chaining, then checks the remaining anchors in the aligned region to discover potential inversions. We benchmark invMap on simulated datasets across different genomes and sequencing coverages, experimental results demonstrate that invMap is more accurate to locate aligned regions and call SVs for inversions than the competing methods. The real human genome sequencing dataset of NA12878 illustrates that invMap can effectively find more candidate variant calls for inversions than the competing methods.

**Availability and implementation:**

The invMap software is available at https://github.com/zhang134/invMap.git.

## 1 Introduction

Mapping sequences to a reference genome is a fundamental bioinformatics task in most genome sequence analysis pipelines (Döring *et al.* 2008, Yu *et al.* 2023). Since the birth of the first high throughput sequencing platform, numerous mapping methods have been proposed in the bioinformatics research community (Xin *et al.* 2013, Chen *et al.* 2023, Wei *et al.* 2023). Long read sequencing, developed by Pacific Biosciences (PacBio) and Oxford Nanopore Technology (ONT), is becoming more popularity with reduced end-to-end sequencing times, improved raw read accuracy, lower costs of adoption and ease of portability (Rhoads and Au 2015, Wei and Zhang 2017). Importantly, these innovative technologies increase read length to over thousands of base-pairs (bp), which can promote various cutting-edge genomic studies. Compared to next-generation sequencing, long reads produced by PacBio or ONT have higher error rates (∼15% versus 1%) with different types of errors (indels rather than substitutions) (Wei *et al.* 2017, Wei and Zhang 2018). With such long noisy reads, mapping has again become a central bioinformatics challenge.

Over the past decades, a number of available approaches have been designed for mapping long noisy reads against the reference genome. State-of-the-art long read mappers include BLASR (Chaisson and Tesler 2012), BWA-MEM (Li 2013), rHAT (Liu *et al.* 2015), LAMSA (Liu *et al.* 2016), GraphMap (Ivan *et al.* 2016), NGMLR (Sedlazeck *et al.* 2018), minimap2 (Li 2018), lordFAST (Haghshenas *et al.* 2018), DuploMap (Prodanov and Bansal 2020), Winnowmap2 (Jain *et al.* 2022), smsMap (Wei *et al.* 2020), lra (Ren and Chaisson 2021), and kngMap (Wei *et al.* 2022). A majority of those existing methods adopt the classical seed-chain-extend mechanism (Wei and Zhang 2015, Sahlin *et al.* 2023), which mainly works in three stages. First, each query read is broken down into *k*-mers or smaller segments (called seeds), then these seeds are used to find matched locations in the reference genome through an indexing technique, each match is referred as to an anchor. Second, alignment skeleton or chain is formed by selecting a set of co-linear, nonoverlapping anchors on the reference genome that has the highest score among all such sets using colinear chaining algorithm (Ohlebusch and Abouelhoda 2006). Third, during the extension stage, the detail bases-to-base alignment of the entire query read is obtained by performing pairwise sequence alignment for gaps between pairs of consecutive anchor matches.

With the development of long sequencing technologies, the maximum and N50 read lengths can respectively achieve to 4 Mbp and 100 kbp (van Dijk *et al.* 2023), which are orders of magnitude longer than Illumina and can easily span the breakpoints of various structural variations (SVs), such as inversions, deletions and insertions in the human genomes. The long read lengths can significantly help applications like *de novo* assembly and SVs calling (Akbarinejad *et al.* 2021). Related studies have reported that each human has around 20 000 SVs on average (Khan *et al.* 2023, Zheng and Shang 2023). This may greatly influence the long-read mapping due to that most state-of-the-art mappers are designed for SV-free reads. Hence, the current mapping research demands the development of better algorithmic aligners to deal with SVs reads efficiently.

As far as we know, among existing mapping algorithms, LAMSA (Liu *et al.* 2016), DuploMap (Prodanov and Bansal 2020), lra (Ren and Chaisson 2021), and Winnowmap2 (Jain *et al.* 2022) are specifically designed for handling SVs reads. LAMSA (Liu *et al.* 2016) starts to extract a series of seeding fragments at every *FI* bp (each of the fragments is *FL* bp long) from each read and finds the approximate matches of the fragments on a reference genome. Then, it constructs a direct acyclic graph and selects a set of alignment skeletons by performing a sparse dynamic programming strategy. Each skeleton consists of a series of co-linear events and/or non-co-linear events. Lastly, LAMSA classifies the gaps within the skeletons to four categories (match, duplication, deletion and insertion) and fills gaps to address the breakpoints of SVs. DuploMap (Prodanov and Bansal 2020) first constructs a graph based on the known segmental duplications, and identifies reads that overlap segmental duplications in the cluster. Then, it filters out unlikely aligned locations by developing a longest common subsequence-based strategy, which utilizes *k*-mers that are unique to a particular alignment location. Lastly, for reads that have more than one possible alignment location, DuploMap uses a paralogous sequence variant-based approach to determine the most likely alignment location. lra (Ren and Chaisson 2021) first filters the minimizers and applies a rough and fine two-step clustering strategy to find chains between the query and target, then, the banded alignment is used to obtain the pairwise alignment with a concave-cost gap penalty, which is helpful for modeling the SVs. Winnowmap2 (Jain *et al.* 2022) is a specialized method for accurately mapping long reads to repetitive regions in the reference by introducing the idea of minimal confidently alignable substrings (MCAS), which are minimal-length read substrings that align end-to-end to a reference with mapping quality (confidence) score above a user-provided threshold. Winnowmap2 initially computes MCAS from a subset of equally spaced starting positions (every 1000th base for HiFi and every 2000th base for ONT sequences). In computing MCAS, the alignment search space is reduced by utilizing the weighted-minimizer sampling algorithm (Jain *et al.* 2020). Next, Winnowmap2 extracts anchors that are colinearly chained in each MCAS alignment. Finally, the banded alignment is performed to fill the gaps between pairs of consecutive anchors.

During the seed-chain-extend workflow, the chaining procedure plays an important role in determining the overall throughput and scalability of read alignment, as it can eliminate many locations that would result in an incorrect mapping (Wei *et al.* 2017, Yan *et al.* 2021). In addition, how to select candidate chains determines whether a potential SVs can be detected or not. Most existing methods prioritize the candidate aligned regions based on their chaining scores and performs the final alignment for the top *N* regions. For large SVs (such as inversion), some modern methods can detect them since the SVs region is in the top *N* chains. However, when the inversion length has small size (e.g. <500 bp) or more sequencing errors occur in the SVs regions, there are fewer matched *k*-mers, leading it to be hard to detect such SVs.

In this study, we seek to address this limitation by developing a new long-read mapping method that is sensitive to the presence of inversion with various sizes. Since the mapped positions of *k*-mers from the inversion part of the read are close to those of *k*-mers from SV-free part, we propose a two-step long read alignment strategy (referred to as invMap) with prioritized chaining, which separately deals with the main chain and potential inversion-chain in the candidate aligned region. In this strategy, invMap first searches the main chain with the highest score with a specifically designed chaining strategy, then, preferentially finds the shorter chain from the remaining anchors in the candidate aligned region. The main chain generated in the first stage guarantees that the region, containing the maximum number of concordant anchors, can be selected as the candidate region for alignment. The short chain selected in the candidate region can make sure that the potential inversion events can be detected, even they have small size or occur in the boundaries of the aligned region. Using experimental results from a series of simulated and real human genome sequencing benchmarks, we show that invMap aligns consistently with the most accurate aligned location across different genomes, achieves higher accuracy for the discovery of deletions, insertions and inversions. The analysis of NA12878 illustrates that invMap can effectively find more candidate variant calls for inversions, when compared to other commonly used long-read mappers.

## 2 Materials and methods

**Figure 1.**
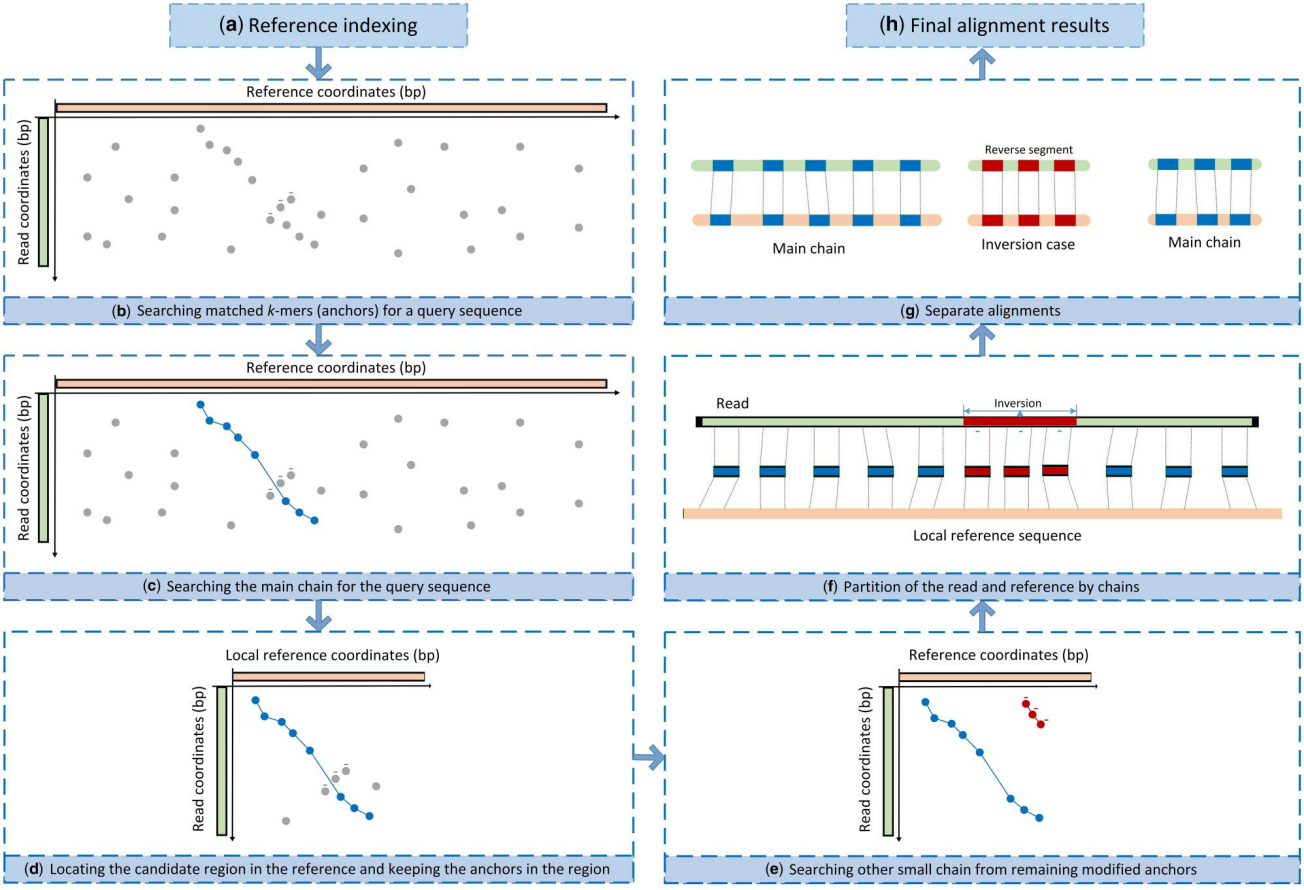
A schematic illustration of the invMap algorithm. (a) Constructing the index from the reference genome, which is used to retrieve exact matches. (b) Establishing the distribution of the matched *k*-mers (also called anchors) between the query sequence and the reference. (c) Searching the main chain (marked with blues node and edges) with a specifically designed chaining procedure. (d) Locating the aligned region in reference covered by the main chain while keeping all anchors in the aligned region. (e) Modifying the reverse anchors (they are nonlinear to those in the main chain) to linear cases and reperforms the chaining on them. In this case, besides the main skeleton, there exists one small chain (marked with red nodes and edges) in the remaining anchors. (f) An illustration of the partition of the read and local reference sequence by chains. In this case, there exists an inversion (marked by red color) in the middle of the read. (g) As the existence of an inversion, the read is separated into three parts. In each part, invMap performs the detail base-to-base alignment with dynamic programming to fill the gaps. (h) After filling the gap, each of chains can be transformed into a valid alignment for the corresponding read part, invMap outputs the final alignments of the three parts in SAM format.

The main motivation of invMap mapper is to effectively deal with potential inversions with various sizes spanned by long noisy reads while keeping high mapping accuracy. invMap first locates the aligned region with a specifically designed scoring method for chaining, then checks the remaining anchors in the aligned region to discover potential inversions. By transforming the non-co-linear anchors to co-linear cases, invMap can find the inversion events even with small size. Overall, invMap mainly consists of four components. It first indexes the reference genomes for fast retrieval. Next, it finds the main alignment chain with a high quality and locates the candidate aligned region in the reference covered by the main chain. Thirdly, invMap modifies the nonlinear anchors occurring in the aligned region to linear ones and identifies small new chains to detect potential inversions. Finally, invMap fills the gaps in each chain to obtain the detail base-level alignment for the whole query read. Figure 1 describes the schematic illustration of the invMap method.

### 2.1 Indexing the references and searching matched *k*-mers

In principle, any state-of-the-art indexing technique can be applied to enhance the search efficiency in the reference genomes with large size for a query *k*-mer. Based on related benchmarking studies (Wei *et al.* 2020, Wei *et al.* 2022), currently, two index techniques are widely used in long-read mappers: BWT-FM and hashing table. Both index techniques have their own advantages. BWT-FM combines the Full-text index in Minute space (FM) (Ferragina and Manzini 2000) and Burrows-Wheeler Transform (BWT) (Burrows 1994) together for fast retrieval. A remarkable advantage of BWT-FM index is that it supports exact pattern matching of arbitrary lengths within small memory space. Hash table allows linear time to find the positions in the genome when a certain *k*-mer is given. Compared with BWT-FM, the indexing speed based on the hash table is faster, but it needs more memory usage. In consideration of runtime efficiency, invMap uses hashing table implemented in *minimizers* (Li 2016) to construct the index for the reference genomes.

With the hash index of the reference, invMap extracts all *k*-mers for one query read and retrieves the matched *k*-mers in the reference genomes, as shown in Fig. 1b. Each of the matches (also called anchors) can be denoted as a 4-tuple *match_i_* = (*pr_i_*, *pg_i_*, *dr_i_, dg_i_*), where *pr_i_* is the matching position on the read for *match_i_*, *pg_i_* is the position on the genome, *dr_i_* and *dg_i_* are respectively the direction (1 denotes plus strand and 0 minus strand) of *match_i_* on the read and reference. All anchors are sorted in ascending order by their positions on reference genomes and then on the query read.

### 2.2 Building the main chain of alignment

The main chain of the alignment is defined as a subset of anchors among all anchors and the paired segments (also called gaps) partitioned by anchors. The best chain should select a group of concordant anchors that has the highest score among all such groups based on chaining. In consideration of the potential inversions and high sequencing errors contained in reads, in this study, we elaborately design a scoring strategy based on dynamic programming to find the chain of alignment with a high quality. Due to the fact that SVs do not occur as frequently as that of small co-linear variants such as SNPs and small indels (Mills *et al.* 2011), invMap assumes all anchors as SV-free at first, and computes the score for each anchor as follows.

Initially, the score of each anchor is set with 0. Then, let *score*(*i*) be the maximal chaining score up to the *i*-th anchor, *score*(*i*) can be calculated with the following dynamic programming equation:


(1) score(i)=max{score(j)+1},j∈precursor(i)

where 1 is the reward score between two anchors, *precursor*(*i*) is the precursor set of the *i*-th anchor, which is defined as the set of anchors whose strand direction and distance with the *i*-th anchor satisfy following 4 conditions: *dr_j_* == *dr_i_* & *dg_j_* == *dg_i_* & *pr_j_*-*pr_i_* ≤ *d* & *pg_j_*-*pg_i_* ≤ *d*, where the constraint parameter of *d* describes the maximum distance allowed between two anchors in the true alignment for a read. The strand direction conditions in Equation (2) can guarantee that anchors within the same chain are from the same strand, facilitating the detection of inversion event. For long noisy reads, we assume the error rate is 15%, so we set *d *=* *1.15 × *len*(*r*), which is a variable value depending on the length of the query read *r*. With the setting of *d*, it can avoid calculating unnecessary anchors in Equation (1) and significantly speed up chaining procedure.

After calculating the scores for all anchors, invMap selects the optimal path connecting *m_start_* and *m_end_* which maximizes the highest increased score as the main skeleton of alignment with the following equation:


(2) $$\arg\underset{{\mathrm{match}}_{\mathrm{start}},{\mathrm{match}}_{\mathrm{end}}}{\max}\left\{\mathrm{score}\left({\mathrm{match}}_{\mathrm{end}}\right)-\mathrm{score}\left({\mathrm{match}}_{\mathrm{start}}\right)\right\}$$

where *match_start_* ∈ *precursor*(*match_end_*), then the path starting from *match_start_* and ending at *match_end_* forms the main chain of alignment for the query read.

### 2.3 Finding the new chain for inversion SV

With the main chain, invMap locates the aligned region in the reference covered by the main chain, and keeps all anchors occurring in the aligned region. Compared with deletion and insertion (Heller and Vingron 2019), inversion event is more difficult to be detected since the anchors in inversion region is nonlinear to the anchors in the main chain. Thus, invMap tries to find a potential inversion by converting the nonlinear anchors to linear ones, and then searches other chains among the nonlinear anchors.

First, invMap uses a modified 3-triple *m_i_* = (*mpr_i_*, *mpg_i_*, *r_i_*) to present each remaining anchor in the aligned region for dealing with co-linear and nonlinear events:


(3) {mpri={pri, if dri==dgilenr-pri, othersizempgi=pgiri={'+', if dri==dgi′-′, otherwise

One can see that the main differences of *m_i_* with *match_i_* are the positions on the query sequence and direction donation. If *dr_i_* and *dg_i_* have the same direction, then *r_i_* = “+”, *mpr_i_* = *pr_i_*, otherwise, *r_i_* = “−”, *mpr_i_* = *len*(*r*) – *pr_i_*, where *len*(*r*) is the read length, *r_i_* denotes the strand consistency: the “+” symbol indicates that this anchor from the read is matched to the plus direction of the reference, while “−” to the minus direction of the reference. The modified *m_i_* can change the nonlinear chain to the linear case, which can significantly facilitate the detection of inversions.

**Figure 2.**
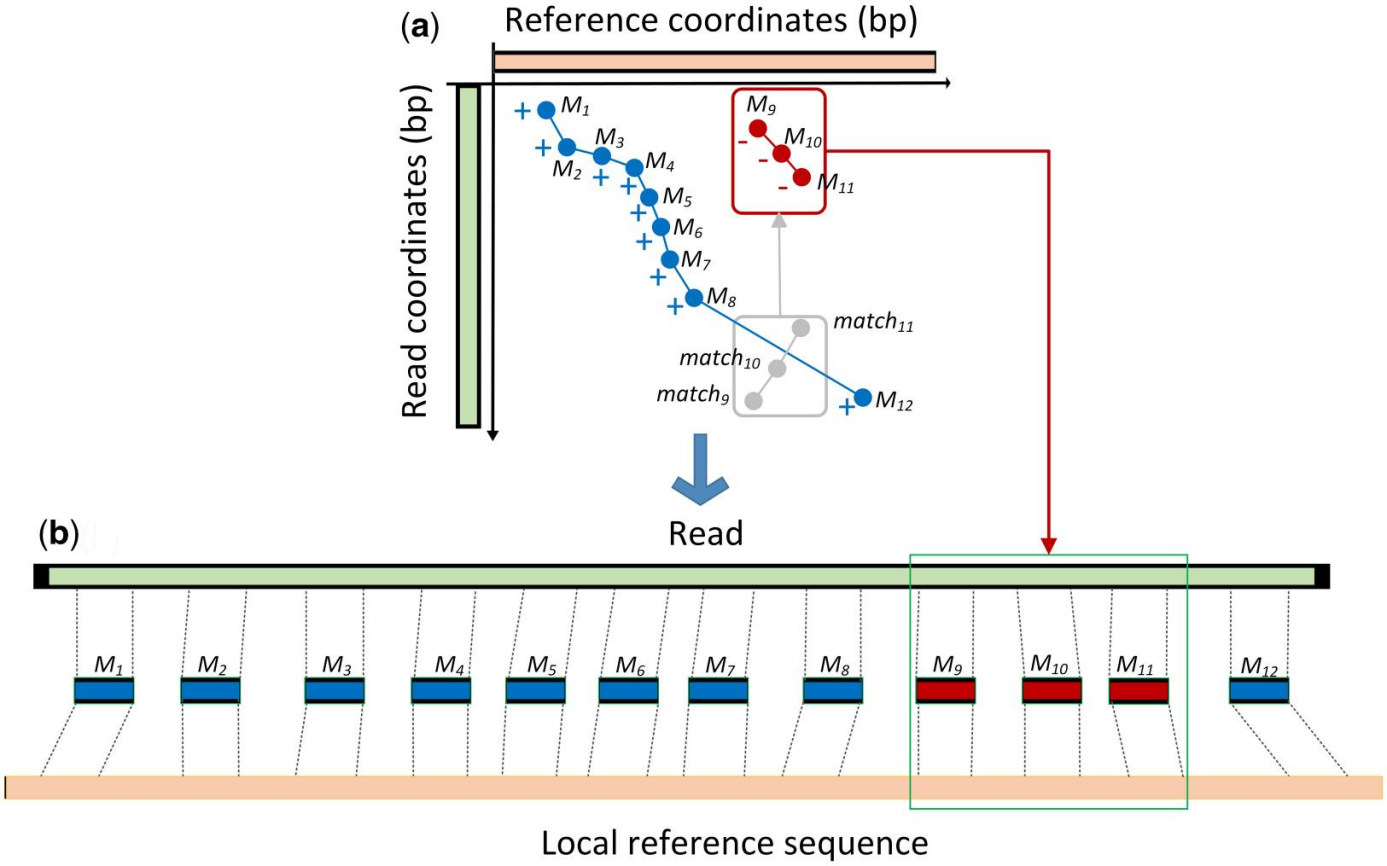
A schematic illustration of finding inversion event. (a) The main chain (marked by blue dots) and shorter chain (marked by red dots). The main chain consists of nine anchors: *M_1_*, *M_2_*, *M_3_*, *M_4_*, *M_5_*, *M_6_*, *M_7_*, *M_8_*, and *M_12_*. The shorter chain consists of three anchors: *M_9_*, *M_10_*, and *M_11_*. The “−” symbol around the anchor indicates that this anchor from the read is matched to the reverse strand of the reference, otherwise, to the plus strand. The original three matches (*match_9_*, *match_10_*, and *match_11_*) come from the inverse part in the read, therefore, they are noncolinear compared with other matches in the main chain, but they are transformed to the linear anchors (*M_9_*, *M_10_*, and *M_11_*) by using Equation (3), as the result, they can scored by Equation (1), and can be detected by researching with the highest increased score. (**b**) An illustration of the partition of the read and local reference sequence by the two chains in (**a**). In this case, the shorter chain is an inversion event since that all the anchors (*M_9_*, *M_10_*, and *M_11_*) in the chain come from the reverse strand of the read.

Then, invMap calculates the scores for remaining anchors that are not included in the main chain. Like finding the main chain in previous step, a new shorter chain with the highest increased score is selected among the remaining anchors. If the direction is “−” for all anchors in the new chain, it indicates that these anchors come from the reverse strand of the query. Since the new chain is commonly shorter than the main chain, it is considered as an inversion event. Figure 2 depicts a detail example of how to find the inversion. The main chain consists of blue anchors (*M_1_*, *M_2_*, *M_3_*, *M_4_*, *M_5_*, *M_6_*, *M_7_*, *M_8_*, and *M_12_*) in Fig. 2a represents the main chain built by the previous step. The three anchors (denoted by *match_9_*, *match_10_*, and *match_11_*) are not included in the main chain. Since they (marked by “−”) come from the reverse strand of the query, so they are noncolinear to the anchors in the main chain. Based on Equation (3), *match_9_*, *match_10_*, and *match_11_* are modified to *M_9_*, *M_10_*, and *M_11_*, which become concordant to those in the main chain. Then *M_9_*, *M_10_*, and *M_11_* are scored and selected as the new chain of inversion. Finally, the main and shorter chains partition the read and reference into different paired segments, which are shown in Fig. 2b.

In consideration of the fact that SVs are large genomic alterations, where large is typically defined as encompassing at least 50 bp (Mahmoud *et al.* 2019). Therefore, based on the analysis above, the new shorter chain is categorized into the inversion event if it meets the following two conditions: (i) at least contains three anchors, and simultaneously, (ii) the paired segments covered by these anchors are longer than 50 bp. Finally, for all chains, the pairwise sequence alignment is performed to obtain the detail base-to-base alignment, which is discussed in the following step.

### 2.4 Alignment

In order to generate the detail alignment, invMap applies dynamic programming-based alignment for gaps between pairs of consecutive anchor matches in a chain. For the inner paired segments (flanked by two adjacent anchors) of the chains, global alignment is directly performed to obtain the detail alignments. For the outer boundaries, i.e. the paired segments at the beginning and ends of the chains, which can be also considered as gaps with only one anchor. For each of the boundaries, invMap assumes that the regions in the read are SV-free, and directly extends the anchor by global algorithm. invMap applies the KSW2 library (Li 2018, Suzuki and Kasahara 2018) for computing the global pairwise alignment. The KSW2 is a library for aligning a pair of biological sequences based on dynamic programming, it can greatly accelerate the base-level alignment with the help of SSE2 and SSE4.1 parallelization. The entire read alignment is accomplished by integrating the anchors and the alignments of the gaps.

### 2.5 Additional processing

**Figure 3.**
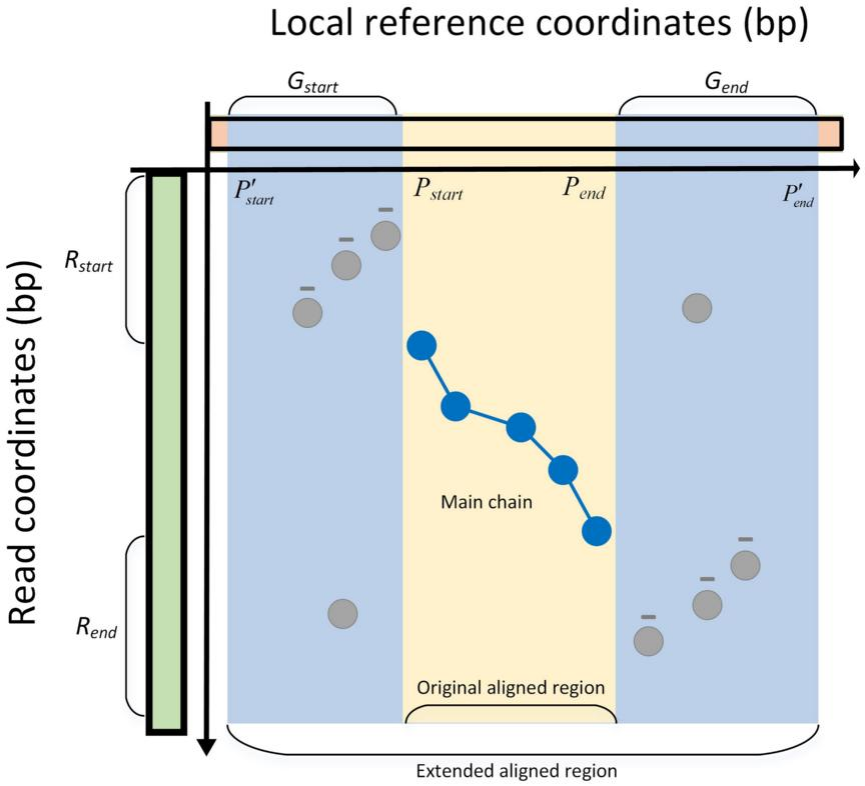
A schematic illustration of the extending procedure in invMap. The chain colored with blue nodes and edges is the main chain. The reference colored by yellow is the original region (starting from *P_start_* to *P_end_*) covered by the main chain. Inversions, at the beginning and ending parts of the read, are not covered by the main region. In these cases, invMap extends the aligned region so that the anchors from the inversion regions can be captured. The final extended aligned region (starting from $p_{\mathrm{start}}^{,}$ to$\;p_{\mathrm{end}}^{,}$) is colored by the blue background.

All the operations in searching the inversion chain focus on the remaining anchors in the aligned region covered by the main chain. However, in some cases, some inversions are in the outer boundaries of the main chain, i.e. the staring or ending parts of the read, which are not covered by the main chain. To deal with these situations, invMap extends the aligned region so that the anchors in the inversion part can be captured. To be more specific, let *R_start_* and *G_start_* respectively denote the prefix of the read and the reference prior to the first anchor in the main chain, and the *len*(*R_start_*) be the length of *R_start_*. invMap extends the starting position (*P_start_*) of the original aligned region to 1.2*len*(*R_start_*) if *len*(*R_start_*) > 50 bp, i.e. *P’_start_* = *P_start_* − 1.2*len*(*R_start_*). Therefore, the anchors can be captured if they are from the inversion region at the beginning of the query read. Similarly, the suffix of the read and the reference following the last anchor in the main chain can be processed in the same fashion. A toy example of such cases is illustrated in Fig. 3. With these additional extending steps, the inversion occurred in the beginning or ending parts of the query read can be effectively detected and aligned.

## 3 Results

invMap is implemented in the C/C++ programming language and supports multi-thread to take advantage of multi-core computers. It is easy to be downloaded and executed in Linux environment under the MIT license. We evaluate the performance of invMap on both simulated and real sequencing data, and compare it with four state-of-the-art long-read aligners, namely, minimap2 (Li 2018), Winnowmap2 (Jain *et al.* 2022), lra (Ren and Chaisson 2021), and NGMLR (Sedlazeck *et al.* 2018). We exclude LAMSA because it always either crashed or produced malformatted output. We also do not compare against DuploMap because it is not a stand-alone tool but a software pipeline applying various extant mapping tools. All experiments are conducted on a Linux server with dual 32-core Intel(R) Xeon(R) Platinum 8336C CPUs clocked at 2.30 GHz, 128 GB of random-access memory (RAM), an INSPUR 40 TB disk running CentOS 7.5 system. Each mapper is performed using their recommended parameters and 64 CPU threads, the detail running command lines can be found in Supplementary Table S1.

### 3.1 Benchmarking on simulated data across sequencing technologies

First, to evaluate the location accuracy of the chaining procedure designed by invMap, a wide range of synthetic datasets are generated to capture the diversity of sequencing platforms and the complexity of different genomes. We use PBSIM (Ono *et al.* 2012) to simulate PacBio circular consensus sequencing (CCS, also called HiFi) reads and PacBio single-pass reads (CLR) with default settings, PBSIM2 (Ono *et al.* 2021) is applied to simulate ONT reads with recommended parameters. All simulated datasets are generated at coverage level of 20× from three reference genomes with different sizes: *Neisseria meningitidis* (∼2.18 Mbp), *Abortiporus biennis* (∼33.12 Mbp) and chromosome 1 (∼230 Mbp) of GRCH37. Supplementary Tables S2 and S3 report the command line parameters of each simulator and the reference genome information from the NCBI.

For each simulated read, PBSIM and PBSIM2 provide the “true” original location on the reference genome and the strand of the read to the reference genome in that location. Therefore, we have been able to calculate the number of correctly located reads. A read is considered as correctly located if (i) it gets mapped to the correct strand; and (ii) the subsequence on the reference genome the read maps to, overlaps 90% of the length of the “true” mapping subsequence. Unaligned reads and soft- or hard-clipped portions of aligned reads are not taken into account for the location accuracy calculation.

**Figure 4.**
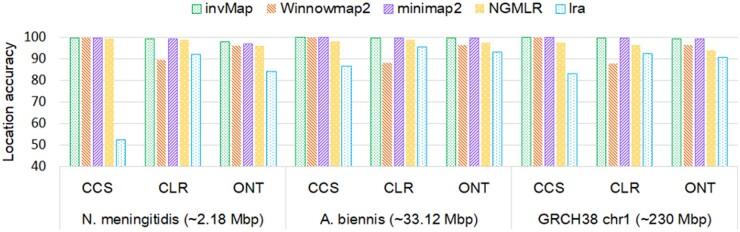
A comparison of location accuracy for five mapping methods on simulated datasets across different sequencing technologies. Genomes are ordered horizontally by genome size from smallest to largest.

The location accuracy is then measured for different mapping algorithms. The mapping results are illustrated in Fig. 4, the detail accuracy values can be found in Supplementary Table S4. Overall, we can observe that invMap achieves the highest location accuracy across all genomes and sequencing platforms. Specifically, invMap’s location accuracy scores consistently stay higher than 99% for CCS and CLR reads, and higher than 98% for ONT reads. When compared to the competing mappers, invMap improves the location accuracy by 0.1%–0.9% over other four aligners on synthetic CCS, CLR, and ONT reads, respectively. The minimap2’s performance is the closest to invMap, it obtains higher location accuracy than Winnowmap2, NGMLR, and lra. It is also worth noting that lra always has the lower location accuracy than other methods for CCS reads and ONT reads across different sizes of genomes, Winnowmap2 gets the lower accuracy than other methods for CLR reads, these phenomena indicate that Winnowmap2 and lra are sensitive to the sequencing technologies, while invMap and minimap2 are robust to sequencing technologies. It is found that all methods perform similar results for a certain type of data across different genome sizes, which indicates that genome size does not have influence on the mapping accuracy. The mapping results shown in Fig. 4 and Supplementary Table S4 demonstrate that the chaining strategy developed in invMap can correctly locate the mapping position for long CCS, CLR, and ONT reads.

### 3.2 Benchmarking on simulated data with inversions

Next, in order to assess the ability of handling with inversions for different mapping approaches, in this experiment, one simulated dataset is generated with different sizes for inversions. Specifically, the SURVIVOR (Jeffares *et al.* 2017) simulator is used for the simulation of inversions in any genome available as the FASTA file. SURVIVOR is firstly applied to generate 100 inversions (the ground truth) for a given reference genome (here, we still apply the chr1 used in previous experiment as the reference), then, the genome sequence containing inversions is taken as the input sequence to PBSIM2 for yielding the final simulated CLR dataset at the coverage level of 40×. With the simulated reads, the breakpoints of the alignments generated by each mapper are evaluated with the ground truth. The length distribution of the 100 simulated inversions is described in Supplementary Fig. S1.

We first extract the inversion reads (totally 3738) and access the number of reads that are correctly aligned by different methods. All the reads are mapped against the original chr1 genome by each algorithm. Each inversion described by the ground truth in the reads can be denoted as a quadruple: ${\mathrm{Inv}}_{i}^{T}=\left({\mathrm{PS}}_{\mathrm{ref}}^{T},{\;\mathrm{PE}}_{\mathrm{ref}}^{T},{\mathrm{PS}}_{\mathrm{read}}^{T},{\mathrm{PE}}_{\mathrm{read}}^{T}\right)$, *i *=* *1,2,…,$N_{\mathrm{Inv}}^{T}$, where ${\mathrm{PS}}_{\mathrm{ref}}^{T}$ and${\;\mathrm{PE}}_{\mathrm{ref}}^{T}$ are respectively the start coordinate and end coordinate of the inversion on the reference genome, ${\mathrm{PS}}_{\mathrm{read}}^{T}\;$and ${\mathrm{PE}}_{\mathrm{read}}^{T}\;$are respectively the start coordinate and end coordinate of the inversion on the simulated read, and $N_{\mathrm{Inv}}^{T}$ (here $N_{\mathrm{Inv}}^{T}$=3738) is the total number of simulated reads that contain inversion. Each breakpoint predicted by a mapper is denoted as a quadruple: *Inv_i_* = (*PS_ref_*, *PE_ref_*, *PS_read_*, *PE_read_*), *i *=* *1,2,…,*N_Inv_*, where *PS_ref_*, *PE_ref_*, *PS_read_*, and *PE_read_* are the elements corresponding to that of a ground truth inversion but predicted by a certain mapper, while *N_Inv_* is the total number of predicted insertions. A predicted inversion *Inv_i_* = (*PS_ref_*, *PE_ref_*, *PS_read_*, *PE_read_*) is considered as correctly mapped if it is covered by the corresponding ground truth inversion ${\mathrm{Inv}}_{i}^{T}=\left({\mathrm{PS}}_{\mathrm{ref}}^{T},{\;\mathrm{PE}}_{\mathrm{ref}}^{T},{\mathrm{PS}}_{\mathrm{read}}^{T},{\mathrm{PE}}_{\mathrm{read}}^{T}\right)$, i.e. [*PS_ref_*, *PE_ref_*] ∩ [${\mathrm{PS}}_{\mathrm{ref}}^{T},{\;\mathrm{PE}}_{\mathrm{ref}}^{T}$] ≠ Ø and [*PS_read_*, *PE_read_*] ∩ [${\mathrm{PS}}_{\mathrm{read}}^{T},{\mathrm{PE}}_{\mathrm{read}}^{T}$] ≠ Ø. In addition, we also evaluate all methods to check their recovery sensitivity and precision. The recover sensitivity is defined as the number of correctly recovered inversions divided by the total number of true inversions. Similarly, precision is defined as the fraction of correctly recovered inversions out of the total number of predicted inversions.

The number of predicted inversions, the number of ground truth inversions being recovered, the recover sensitivity and precision of each mapping algorithm are compared. Table 1 lists the mapping accuracy summary of the five aligners. As can be seen, invMap predicts a total of 3606 inversions, among which 3483 are correctly recovered, and achieves the recover sensitivity to 92.06%. The number of predicted and correctly recovered inversions of invMap is higher than other methods, invMap also has the best recover sensitivity, which is 14.78% higher than that of NGMLR, the closest competitor, and even more (21.70%, 21.35%, and 47.13%) than Winnowmap2, minimap2, and lra, respectively. It is important to note that for lra, the precision value is much higher than the sensitivity because it leaves many of the reads incorrectly unmapped. In that sense, we believe that sensitivity provides a much better measure to compare the tools, even though invMap’s precision is slightly lower than lra (96.58% versus 96.81%), but higher than other methods. These mapping results demonstrate that the two-chaining strategy proposed in invMap can effectively detect inversions and get the alignment results, indicating that invMap succeeds in addressing inversions in these regions by preserving good accuracy where the other tools struggle.

**Table 1.** The number of mapped reads that span SVs breakpoints for different mapping methods.

Methods	invMap	Winnowmap2	minimap2	NGMLR	lra
Total predicted	3606	2946	3137	3130	2416
Correctly recovered	3483	2821	2836	2998	2339
Sensitivity (%)	92.06	75.46	75.86	80.20	62.57
Precision (%)	96.58	95.75	90.40	95.78	96.81

We further investigate the inversions that are not predicted by invMap, and find that such inversions are difficult to recover for all mapping algorithms, which can be attributed to the lack of matched *k*-mers. This usually happens when the inversion regions are short, or contain insufficient anchors due to the sequencing errors. In such situations, these inversion regions can be considered as SV-free parts and directly aligned with the same strategy as in the main skeleton. Supplementary Fig. S2 in Supplementary File shows such an example, we can see that the length of this simulated inversion is 183 bp and contains no anchors (*k*-mer length is 15), all mappers directly align it to the original strand and generate co-linear local alignment with poor quality (i.e. aligned with many indels and substitutions).

In addition, we explore the inversions detected by invMap but do not belong to the ground-true. The inversion similarity and length are shown in Supplementary Table S5. We can see that these inversions’ average identity is around 81.95% with an average length of 1191 bp. Supplementary Figs S3 and S4 illustrate such an example for a new predicted inversion with length of 231 bp, and the inversion identify is around 86.72%. invMap not only gets the alignment for the simulated inversion with length of 227 bp, but also detects another new inversion (#4 record in Supplementary Fig. S3 for invMap), which is not simulated by SRUVIVOR. The base-level alignment for this new inversion is shown in Supplementary Fig. S4. As can be seen, although this new inversion is longer than the simulated inversion (231 vs. 227 bp) and contains many consecutive anchors, Winnowmap2, minimap2, NGMLR, and lra fail to discover the new inversion and report the alignment. These mapping results highlight the invMap’s ability of higher sensitivity for detecting potential inversions than other competing methods.

**Figure 5.**
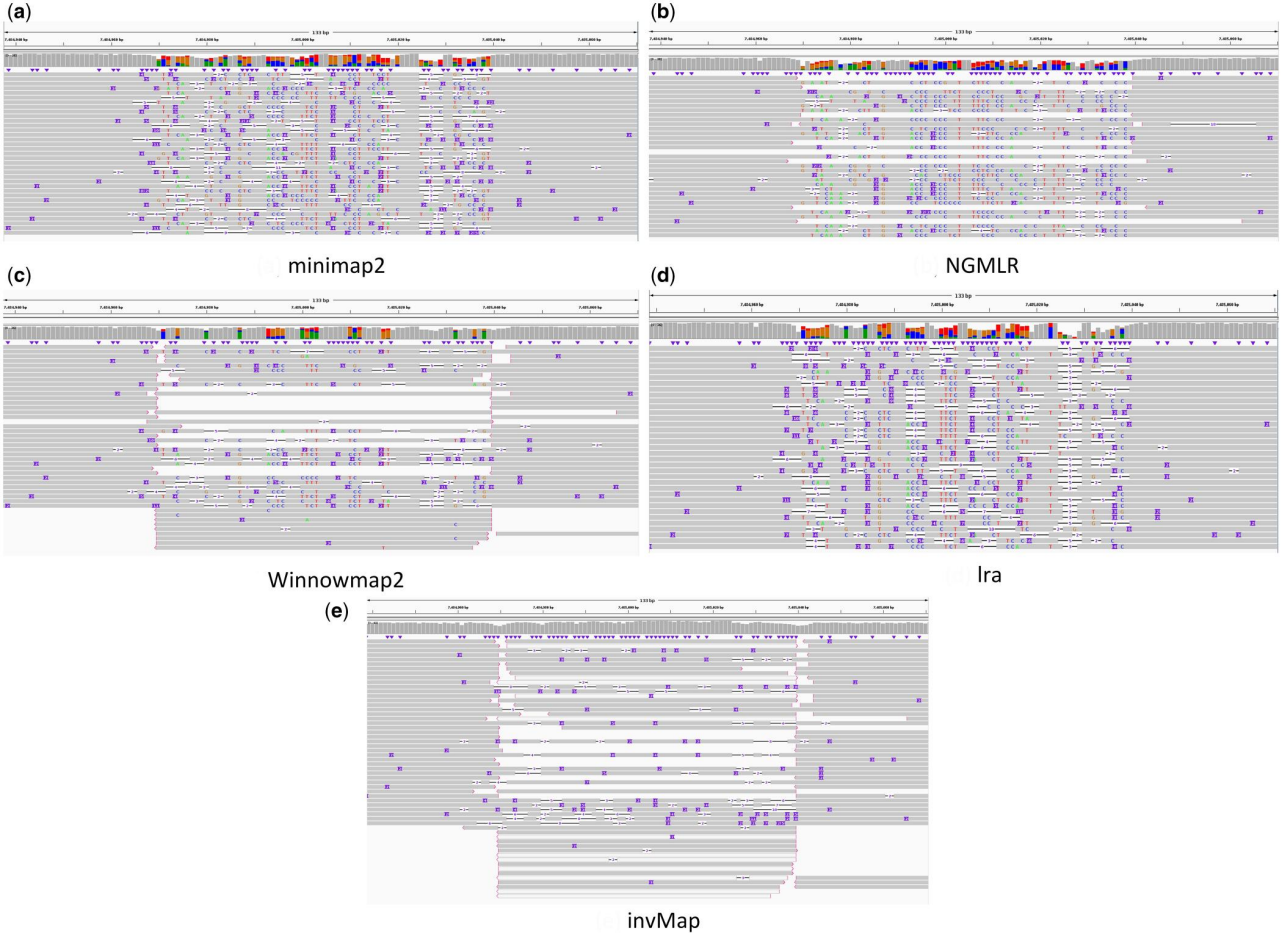
Visualization of alignment pileup near an inversion by using IGV software for different mappers, (**a**): minimap2, (**b**): NGMLR, (**c**): Winnowmap2, (**d**): lra and (**e**): invMap. This inversion starts at locus 7 484 969 and ends at 7 485 038 of chr1. The bar on top of each plot shows mapping coverage. The gray-colored line segments show individual primary read alignments. IGV uses purple markers to indicate presence of indels within read alignments. NGMLR, minimap2, Winnowmap2, and lra show more color bar due to wrong alignments for inversion, while invMap shows expected results in this region.

Then, all simulated reads are mapped and the results of SV calling are provided with SVision (Lin *et al.* 2022) based on the mappings from different tools. SVision is a recently developed variant calling tool based on deep learning for long reads. Figure 5 describes an IGV view of alignments (Thorvaldsdóttir *et al.* 2013) obtained by invMap and four other mapping methods. We can evidently observe that invMap achieves the expected mapping result in this region with most read alignments showing the expected inversion call, while the other tools map more wrong reads, especially for minimap2, NGMLR, and lra, resulting in reduced coverage and poor quality of alignments. These can be largely attributed to the fact that the main chains generated by minimap2, NGMLR, lra, and Winnowmap2 span the inversion region, and the pairwise sequence alignment is forcibly performed in the inversion region. Among the four methods, Winnowmap2 shows the least bias. These mapping results shown in Fig. 5 illustrate the previously discussed limitation of traditional chaining scores to rank candidate chains for inversions. The proposed strategy of rechain in the aligned region implemented by invMap enables correct inversion detection in this case.

When these alignments are used as input to SVision, the output SV call sets are evaluated using SURVIVOR against its own simulated ground truth. As can be seen in Table 2, mappers show variable performance in their ability to detect inversions through their alignments. In comparison, the invMap-SVision pipeline totally calls 125 inversions, and the correctly called number is 82, providing the best recover sensitivity and 2.5%–15.49% improvement over other mappers. This suggests that invMap-SVision pipeline is more suitable for the inversion discovery than other long-read mappers of Winnowmap2, minimap2, NGMLR and lra.

**Table 2.** Comparison of mappers for inversion calling, total number of simulated inversions is 100.

Methods	invMap	Winnowmap2	minimap2	NGMLR	lra
Total calls	125	117	106	130	86
Correct calls	82	75	75	80	71
Sensitivity (%)	82	75	75	80	71
Precision (%)	65.60	64.10	70.75	61.53	82.55

### 3.3 Benchmarking on simulated data with other SVs

In this experiment, we evaluate how well invMap deals with different types of SVs. We apply chr1 to simulate CLR reads at different coverage levels ranging from 10× to 40×. Totally, 900 SVs, including 600 indels and 300 inversions, are simulated by SURVIVOR. Both the SVs simulation and evaluation of variant sets against the ground truth are conducted with SURVIVOR.

**Figure 6.**
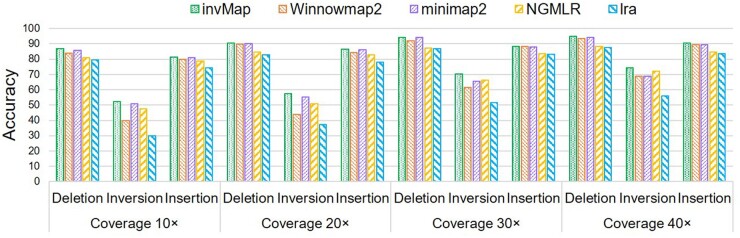
A comparison of accuracy of different SV calls outputted by SVision with five read mapping methods at different coverage levels. Coverage is ordered horizontally by level from smallest to largest.

Figure 6 shows the accuracy statistics of the five mapping tools for each type of SVs at different coverage levels. On the low-coverage level (10×) dataset, invMap has overall the highest number of correctly predicted SVs across all types of SVs. For deletion and insertion SVs, the accuracy shows similar trend, i.e. minimap2 exhibits similar results with invMap, then next comes to Winnowmap2, lra always has the lowest accuracy score. For inversion SV, the accuracy and performance differences for each mapper become more diverse than deletion and insertion. invMap is still the winner followed by minimap2, while NGMLR obtains higher accuracy than Winnowmap2 and lra. When increasing coverage from 10× to 40×, the accuracy of all types of SVs generally improve for all methods as better sensitivity is naturally expected with higher sequencing coverage. However, NGMLR becomes the closest to invMap for inversion detection with the coverage increasing in terms of mapping accuracy. The accuracy of SVs calls for deletion and insertion change slightly when the coverage increases from 30× to 40×, this indicates that the minimum required coverage for deletion and insertion is expected at 30×, but for inversion, the minimum coverage level is 40×. From Fig. 6 we can also observe that, overall, the accuracy for inversion calling is significantly lower than deletion and insertion across all sequencing coverages, demonstrating that the inversion needs to be paid more attention than deletion and insertion. That’s why in this work, we elaborately develop invMap for inversion discovery.

### 3.4 Evaluation using genome in a bottle benchmark

Usually, assessing long-read mappers for SVs calling on real-life sequencing data is challenging since the lack of the gold standard ground-truth information. Fortunately. the Genome in a Bottle (GIAB) Tier1 v.0.6 benchmark set provides a high-quality characterization of SVs in the Ashkenazi cell line HG002 relative to the GRCh37 human reference. One reliable benchmark set of SVs with 2676 high-confidence deletions (Parikh *et al.* 2016) is applied to evaluate the performance of each mapping method. These calls can be freely downloaded at ftp://ftp-trace.ncbi.nlm.nih.gov/giab/ftp/technical/svclassify_Manuscript, which has been widely used as a reliable gold standard set in SVs identification with long reads. Since the SV call set does not contain inversions, therefore, based on some SVs benchmarking studies (Heller and Vingron 2019), we furtherly implant 1000 inversions (produced by SURVIVOR) into the reference genome and align sequencing reads to this altered reference. Implanting an inversion into the reference genome causes the original reads to contain the inverse of the SV that is implanted. With this approach, the performance of discovering inversions can be carefully compared on real sequencing reads for different mappers. Here, one publicly available HG002 long-read sequencing dataset generated by PacBio (ftp://ftp-trace.ncbi.nlm.nih.gov/giab/ftp/data/AshkenazimTrio/HG002_NA24385_son/) is mapped to GRCh37 reference. Similar to previous simulated benchmarks, variants are called using SVision. On the real dataset, invMap’s accuracy is 76.55% (figure not shown), achieves better accuracy compared to minimap2 (75.15%) and NGMLR (73.4%), and the corresponding accuracy scores using Winnowmap2 and lra are 67.10% and 63.25%, respectively. invMap’s ability to deeply discover inversions can yield interesting insights into the dynamics of genomic rearrangements. Our analysis of the HG002 PacBio dataset with invMap (compared to the GRCh37 references) also identifies some high-confidence inversions with many supported reads while other methods fail to identify. Supplementary Figs S5 and S6 illustrate such two examples, invMap successfully produces the inversion alignment for these two regions, while other methods fail to find and obtain the correct alignments. These results highlight the sensitivity of invMap for the detection of inversions better than other competing methods.

## 4 Discussion

Detecting SVs is a common interest for long noisy reads in human genetics. SVs are defined as the genomic variants larger than 50 bp, they have been shown to affect more bases in any given genome than single-nucleotide polymorphisms or small insertions and deletions. Long read, single-molecule sequencing technologies such as PacBio and ONT produce reads with a length of several thousand base pairs. Despite the higher error rate, long-read sequencing offers many advantages for the detection of SVs. Similar to the detection of SVs from short-read data, the first step toward SV detection from long reads is often the alignment of the reads to a reference genome. Across all types of SVs, deletions and inversions are the easiest to detect in the base-to-base alignments with pairwise alignment (Heller and Vingron 2019). However, other kinds of SVs, such as inversion, bring a major obstacle for most extent mapping tools since that the anchors occurs in the inversion regions are non-co-linear to those in the SV-free region. To address this issue, we have developed invMap, specially tailored for inversion detection for long noisy reads. The core step of invMap is that it first locates the aligned region then searches the candidate chains from the inversion regions. By transforming the non-co-linear anchors to co-linear cases, invMap can find the inversion events even with small size.

The huge amount of long-read data generated by PacBio and ONT technologies brings serious challenges to extant mapping methods. Besides mapping accuracy, computational complexity is another important issue that needs to be considered. Supplementary Fig. S7 shows the execution time of the five methods on the simulated datasets applied in experiment 3.3. We can observe that Minimap2 always is the fastest tool followed by lra and Winnowmap2 at all coverages, and invMap is significantly faster than NGMLR. In summary, compared to minimap2, the fastest mappers as far as we know, a drawback of the current implementation of invMap is the higher computational complexity. This can be largely attributed to that invMap extracts all *k*-mers for reference genomes and query reads by default, while other methods, such as minimap2, only choose the minimizers in every sequence of length 20 bp across the genome to reduce *k*-mer searching space during the seeding stage.

## 5 Conclusion

Over the past decades, DNA sequencing technologies such as PacBio and ONT have achieved dramatic improvements in read length, cost and throughput. Sequence mapping, the process of determining the location in the reference genome for each query read, is the first, and often one of the most computation-intensive steps in analyzing genomic datasets. As sequencing technologies continue to increase read length while improving throughput and accuracy, long reads could more frequently span the breakpoints of structural variants (SVs) than that of shorter reads. Accurate and sensitive read mapping of long reads is a prerequisite for accurate and sensitive variant calling in the human genome. Compared to deletions and insertions, inversions are harder to be detected for most extent mapping tools since the anchors in the inversion regions are non-co-linear to those in the SV-free region. In this study, we have developed a novel mapping method invMap, which is elaborately designed for detection of potential inversions. Compared to other commonly used long-read mappers, such as Winnowmap2, minimap2, NGMLR, and lra, experimental results have shown that invMap has higher ability to deal with reads containing deletions, insertions and inversions. We believe that it could be a good choice to incorporate invMap into the developing computational biology pipelines to leverage cutting-edge genomic studies.

## Supplementary Material

btad726_Supplementary_Data
