# Supplementary material for: invMap: a sensitive mapping tool for long noisy reads with inversion structural variants
Source: Bioinformatics. 2023 Dec 7;39(12):btad726. doi: 10.1093/bioinformatics/btad726 (PMC11320709; doi:10.1093/bioinformatics/btad726)
Supplement: btad726_Supplementary_Data [file btad726_supplementary_data.docx]

**invMap: a sensitive mapping tool for long noisy reads with inversion structural variant**

Ze-Gang Wei^1,2^, Peng-Yu Bu^1^, Xiao-Dan Zhang^1^, Fei Liu^1^, Yu Qian^1*^, Fang-Xiang Wu^2*^

1 School of Physics and Optoelectronics Technology, Baoji University of Arts and Sciences, Baoji, 721016, China

2 Division of Biomedical Engineering, Department of Computer Science and Department of Mechanical Engineering, University of Saskatchewan, Saskatoon, SK S7N 5A9, Canada

* Corresponding authors. Email: qianyu0272@163.com; faw341@mail.usask.ca

**Supplementary file**

**Tables**

**Table S1**. Running command lines for each mapping method.

| Programs | Running command lines |
| --- | --- |
| Winnowmap2 | meryl count k=15 output merylDB ref.fa |
|  | meryl print greater-than distinct=0.9998 merylDB > repetitive_k15.txt |
|  | winnowmap -t 40 -W repetitive_k15.txt -ax map-ont ref.fa ont.fq.gz > output.sam |
| minimap2 | minimap2 -t 40 -a genome.fa sequence.fa > minimap2.sam |
| NGMLR | ngmlr -t 40 -r genome.fa -q sequence.fa -o ngml.sam |
| lra | lra index genome.fa |
|  | lra align genome.fa read.fa -t 64 -p s > output.sam |
| invMap | invmap genome.fa sequence.fa > invmap.sam |

**Table S2**. Running command lines and parameter settings of PBSIM/PBSIM2 software for generating simulated datasets.

| Data types | Command lines |
| --- | --- |
| CCS | pbsim --data-type CCS --length-min 1000 --depth 20 --prefix simulated genome.fa |
| CLR | pbsim --data-type CLR --length-min 1000 --depth 20 --prefix simulated genome.fa |
| ONT | pbsim --length-min 1000 --difference-ratio 23:31:46 --depth 20 --prefix ont_pbsim20x genome.fa |

**Table S3**. Reference genomes in simulated experiments.

| References | Download links |
| --- | --- |
| *Neisseria meningitidis* | https://www.ncbi.nlm.nih.gov/datasets/genome/GCF_000002985.6/ |
| *Abortiporus biennis* | https://www.ncbi.nlm.nih.gov/datasets/taxonomy/137743/ |
| Chr1 of GRCH37 | ftp://ftp-trace.ncbi.nih.gov/1000genomes/ftp/technical/reference/phase2_reference_assembly_sequence/hs37d5.fa.gz |

**Table S4**. Accuracy (%) of read location for each mapping method on the simulated CCS, CLR and ONT datasets with different genome sizes.

| Datsets | invMap | Winnowmap2 | minimap2 | NGMLR | GraphMap |
| --- | --- | --- | --- | --- | --- |
| *Neisseria meningitidis* (~2.18 Mbp) | | | | | |
| CCS | 99.77 | 99.75 | 99.77 | 99.35 | 52.33 |
| CLR | 99.23 | 89.52 | 99.21 | 98.79 | 92.13 |
| ONT | 98.03 | 96.08 | 97.06 | 95.91 | 84.09 |
| *Abortiporus biennis* (~33.12 Mbp) | | | | | |
| CCS | 99.95 | 99.96 | 99.93 | 98.29 | 86.63 |
| CLR | 99.63 | 88.29 | 99.63 | 98.72 | 95.60 |
| ONT | 99.60 | 96.40 | 99.60 | 97.39 | 93.22 |
| GRCH38 chromosome 1 (~230 Mbp) | | | | | |
| CCS | 99.96 | 99.96 | 99.96 | 97.30 | 83.07 |
| CLR | 99.76 | 87.80 | 99.75 | 96.57 | 92.41 |
| ONT | 99.45 | 96.26 | 99.45 | 94.03 | 90.66 |

**Table S5**. The length (bp) and identity (%) of inversions detected by invMap but do not belong to the ground truth.

| Ave. length | Min. length | Max. length | Average identity | Min. identity | Max. identity |
| --- | --- | --- | --- | --- | --- |
| 1,191 | 50 | 2569 | 81.95 | 69.75 | 90.19 |

**Figures**


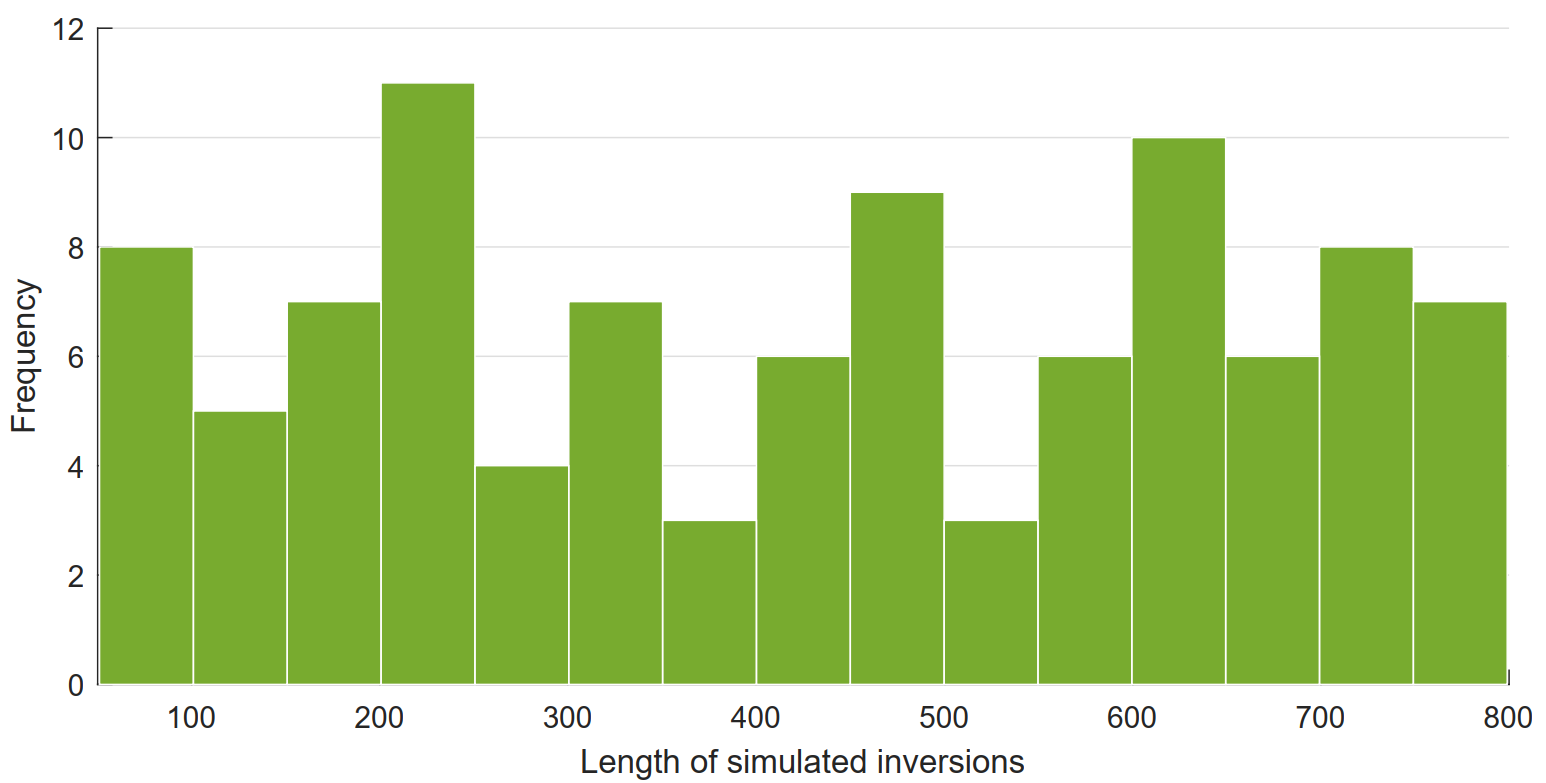


Figure S1. The length distribution of the 100 simulated inversions.


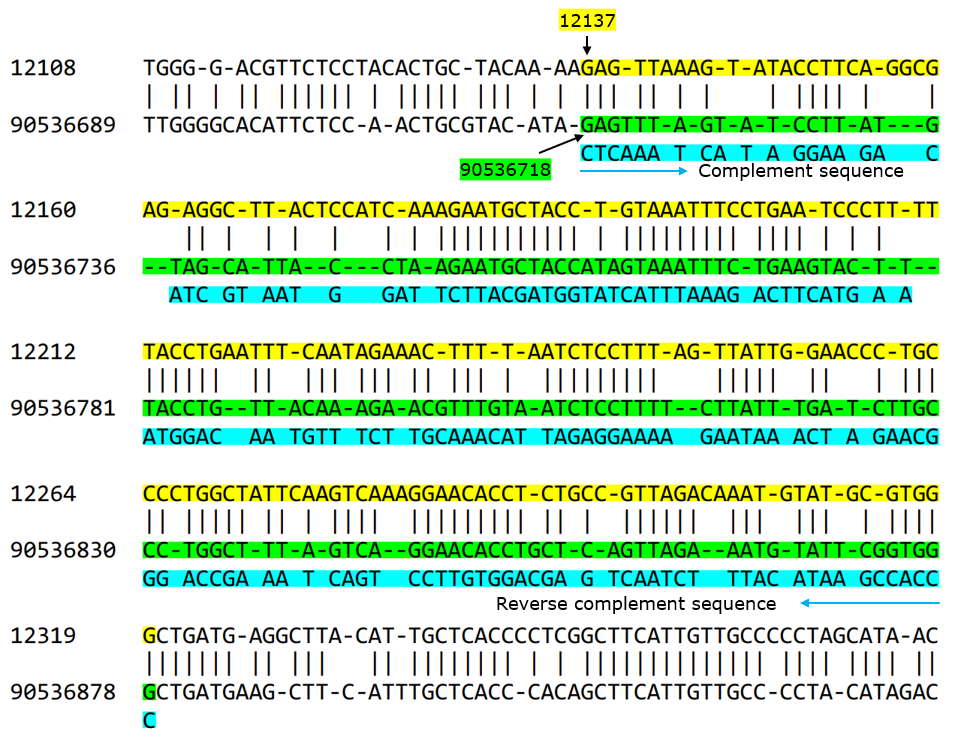

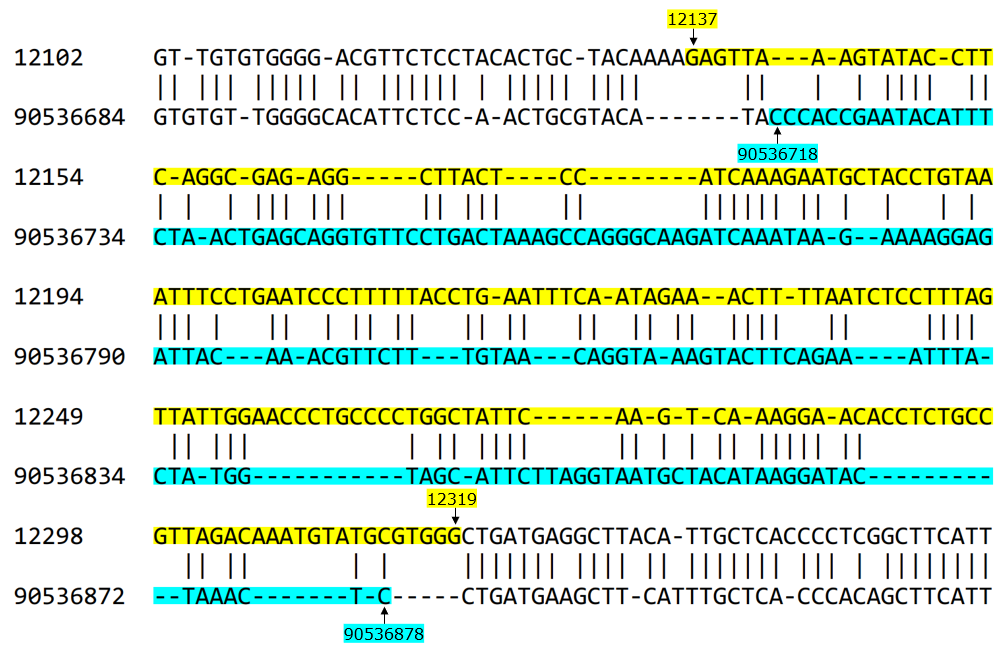


(a) simulated inversion (b) lra


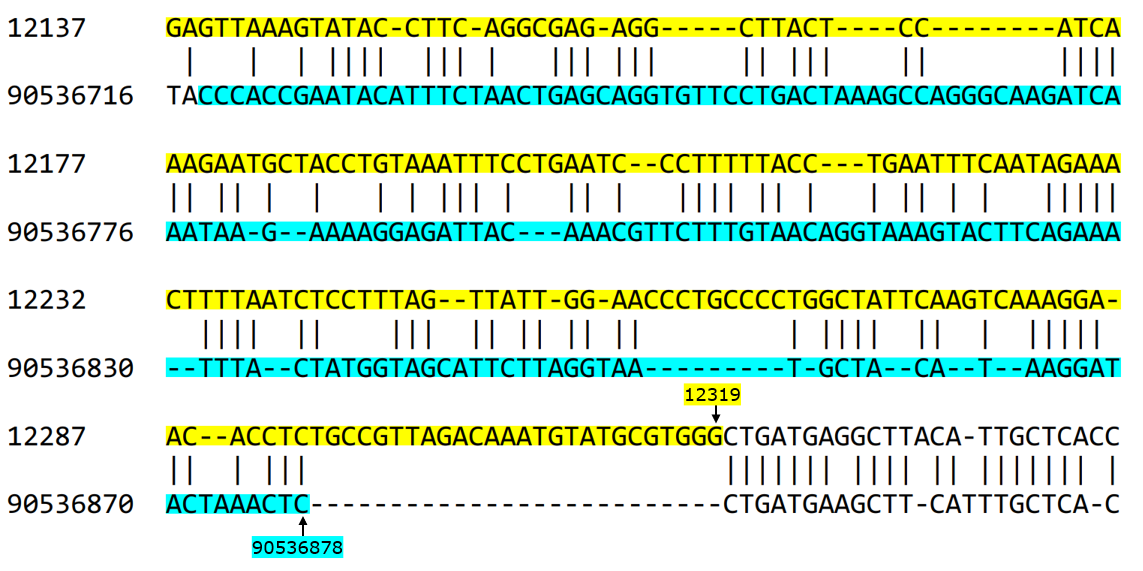

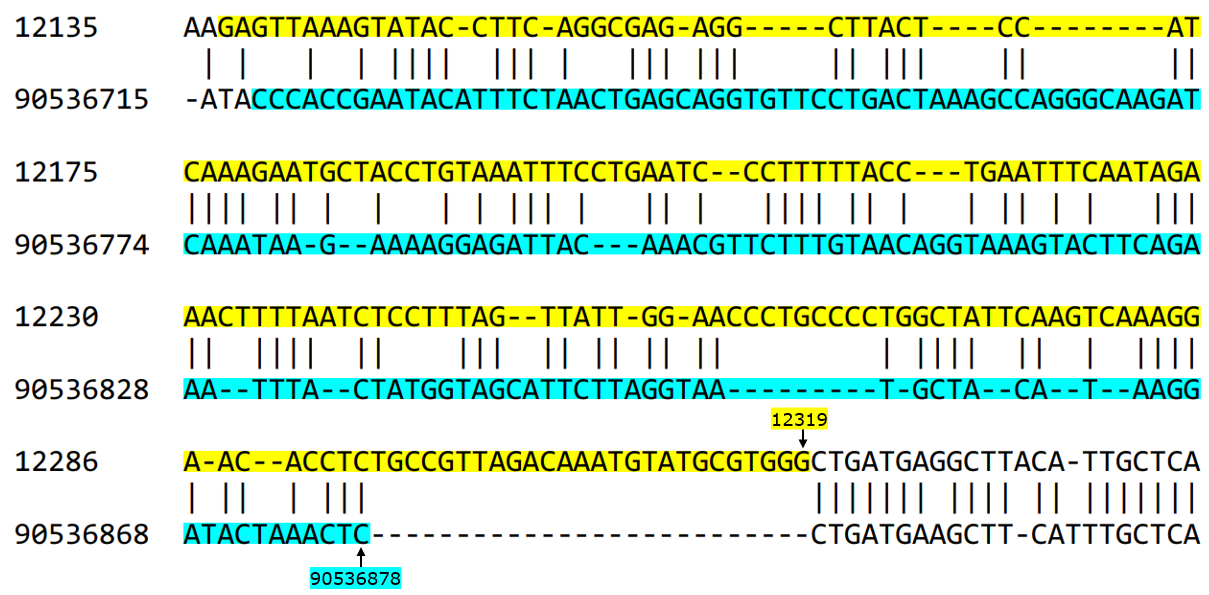


(c) invMap (d) Winnowmap2


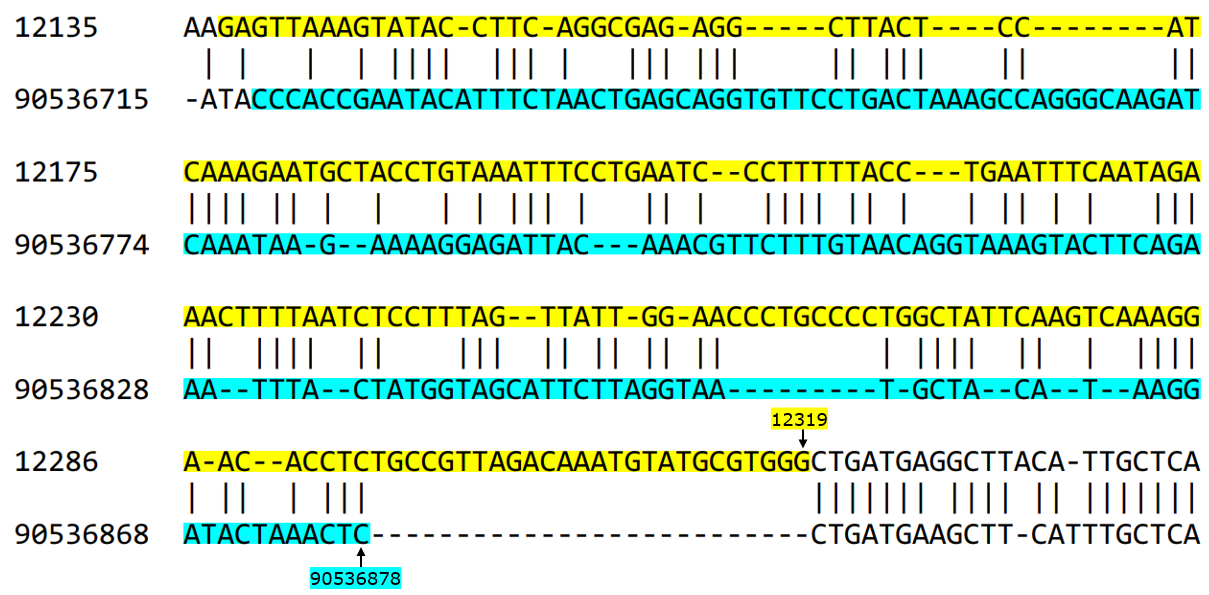

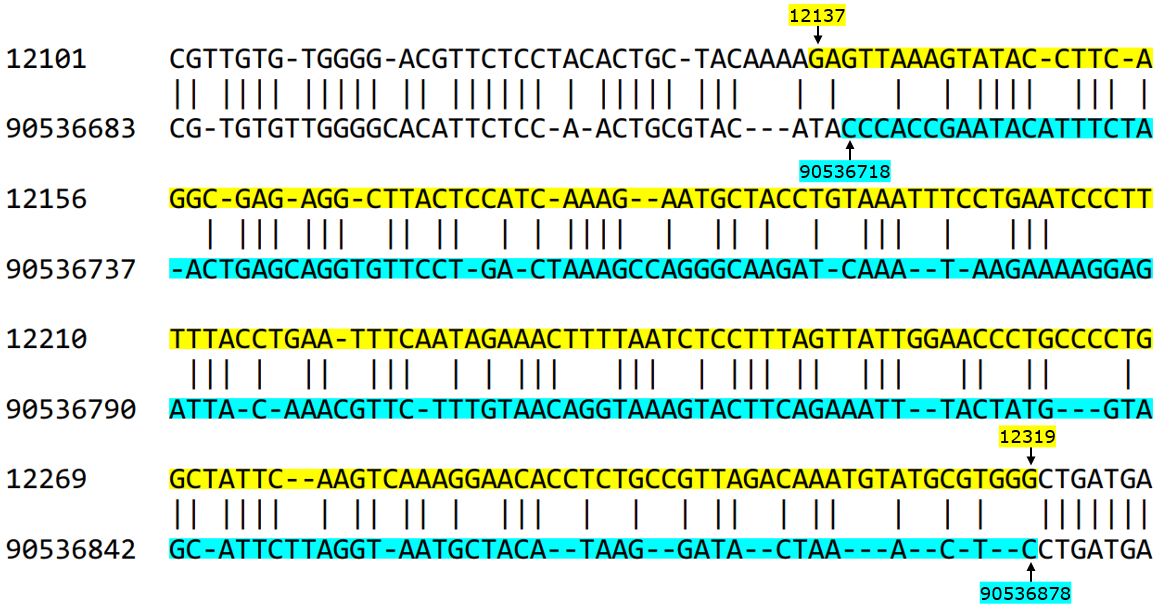


(e) minimap2 (f) NGMLR

**Figure S2**. An example of inversion that are not detected by all mappers. The black arrows with a position are the coordinate positions on the query and reference sequence. (a) The simulated sequence with an inversion is shown in the BLAST-like alignment output format. In this case, the simulated read (13,500 bp, 21.78% error rate) contains a 183 bp inversion (colored by yellow background) which starts from 12,173 to 12,319 positions in the read, the ground truth alignment of the inversion against to the reference (with inversion) is colored by green background, the cyan is the corresponding complement sequence of the reference. The cyan ‘→’ symbol denotes the plus strand of the reference, while ‘←’ symbol denotes the reverse strand. Since there are no anchors (*k*-mer length is 15) in the inversion part, all mappers shown from (b) to (f) directly align it to the original strand and generate co-linear local alignment with poor quality (i.e., aligned with many insertions, deletions and substitutions). It is worth noting that the reference in (a) contains inversions, which are simulated by SURVIVOR, while the reference mapped for each method in figures b-c is the original chr1 genome. Therefore, the subsequence in the reference (colored with cyan background) in Figures (b) to (c) is the reverse complement sequence in Figure (a), which is colored with green background. We can also obverse that NGMLR trends to generate more substitutions than deletions or insertions when compared to invMap, Winnowmap2, minimap2 and lra, this can be attributed to the fact that the scoring procedure in pairwise dynamic alignment is different in each method.


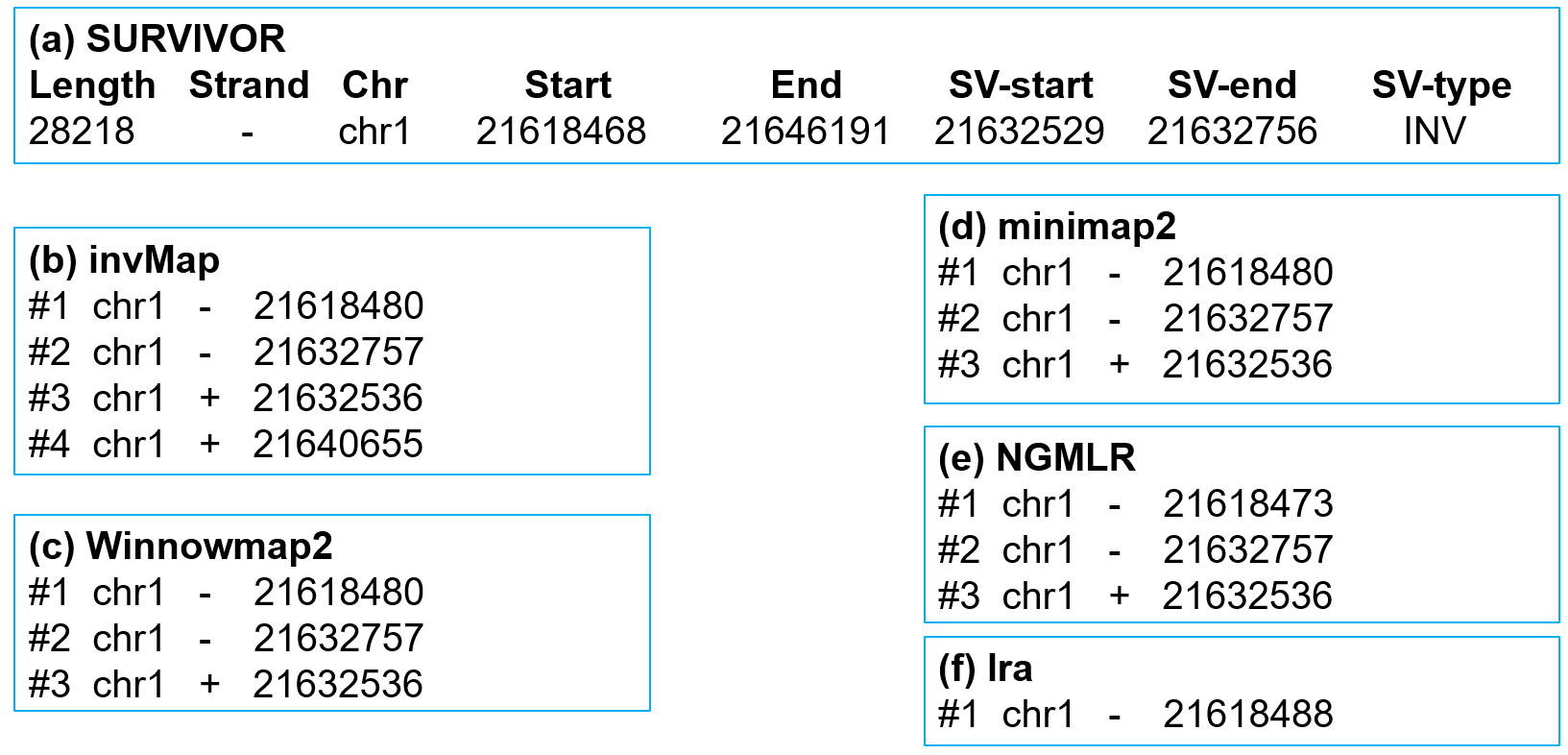


**Figure S3**. An example of the alignment of one simulated read with inversion event. In this case, the simulated read (28,218 bp, 15% error rate) with a 227 bp simulated inversion is aligned. invMap, Winnowmap2, minimap2 and NGMLR suitably align the inversion part of the read with non-co-linear alignment, while lra only generates co-linear alignments for the whole read. **(a)** An integrated inversion event generated by SURVIVOR, all the coordinates (Start, End, SV-start and SV-end) are the positions in the reference. **(b-f)** The SAM records of the read generated by the benchmarked aligners. invMap, Winnowmap2, minimap2 and NGMLR generate three SAM records corresponding to the three parts of read, i.e., the inversion part and the two flanking parts. While lra aligns the read with one SAM record, for the inversion part, it directly aligns it to the original strand and generate co-linear local alignment with poor quality (i.e., aligned with many insertions, deletions and mismatches). It is worth mentioning that invMap not only produces the correct alignments for the simulated inversion part (#2 alignment in the subplot b), but also detects another new inversion (#4 alignment in the subplot b), which is not detected by other methods. The detail bases-to-base alignment for this inversion is shown in Figure S4.


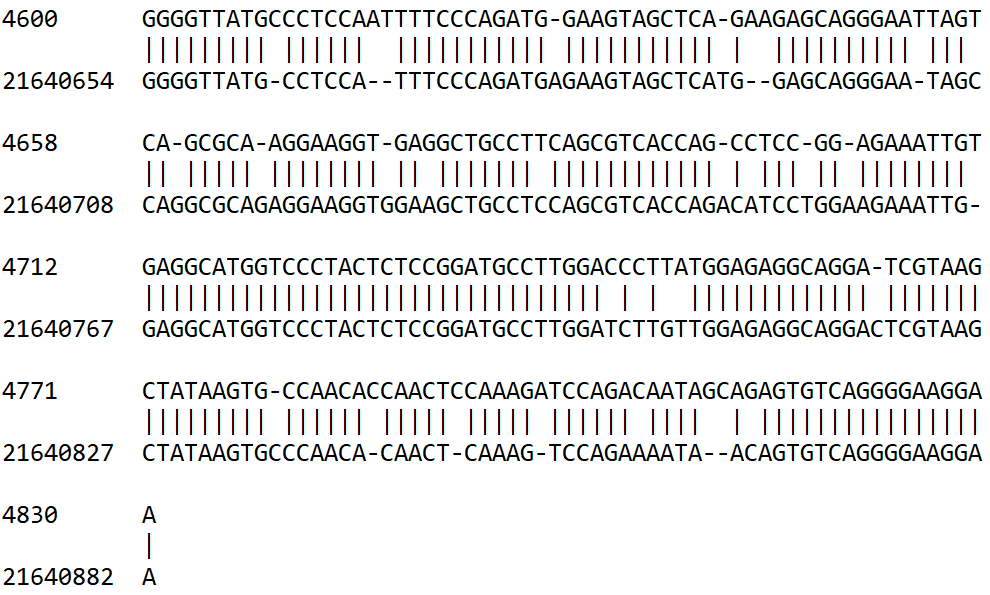


**Figure S4**. The base-level alignment of one inversion, which is only detected by invMap, this inversion is the #4 record in Figure S3(b). The length of this inversion is 231 bp, and the aligned identity is ~86.72%. Nonetheless, other methods cannot detect this inversion.


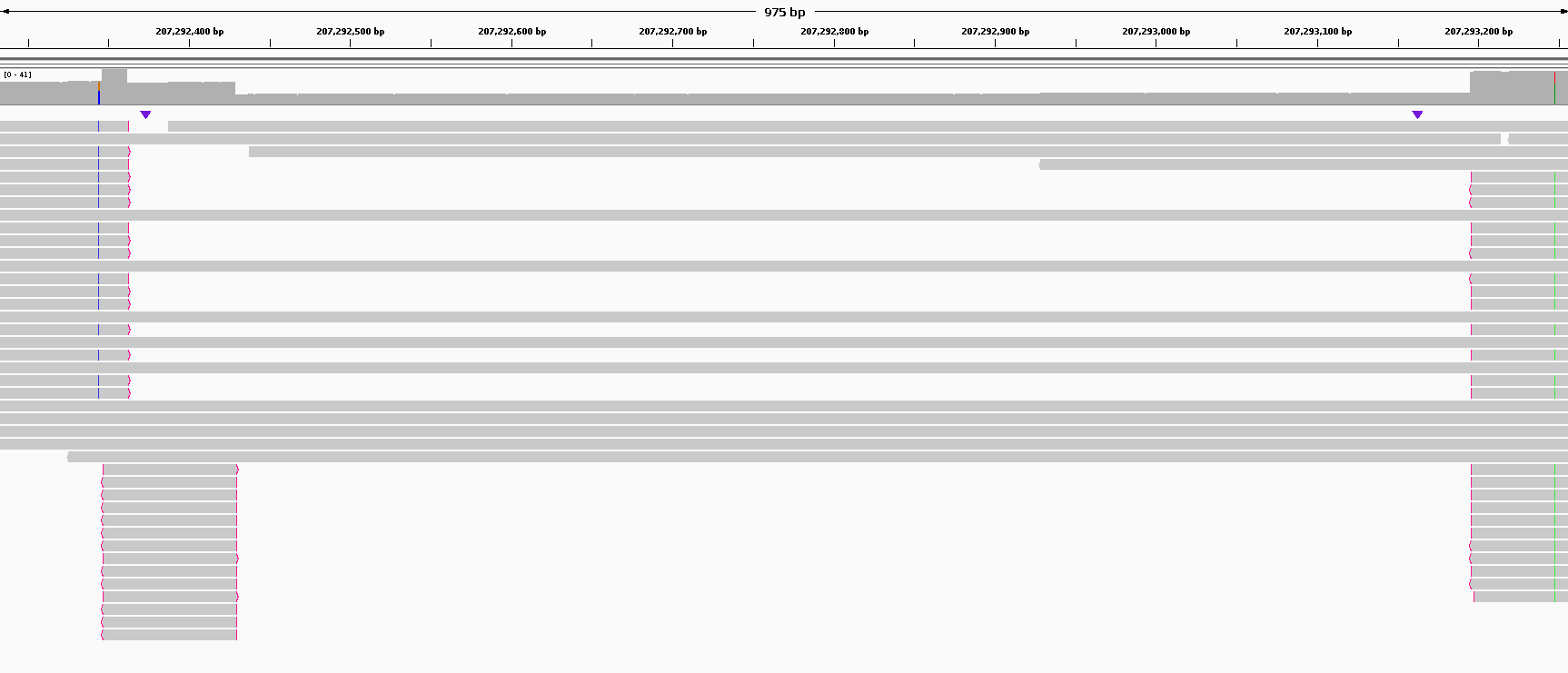

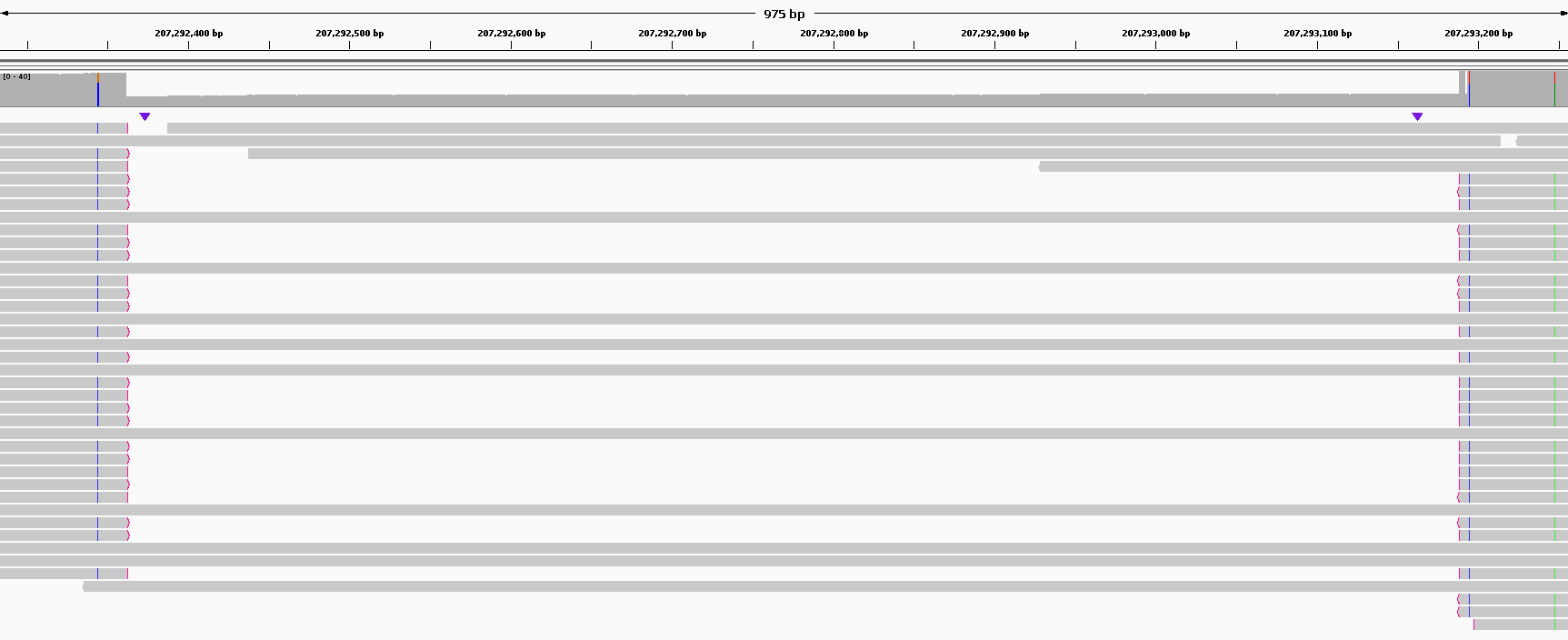


(a) invMap (b) NGMLR


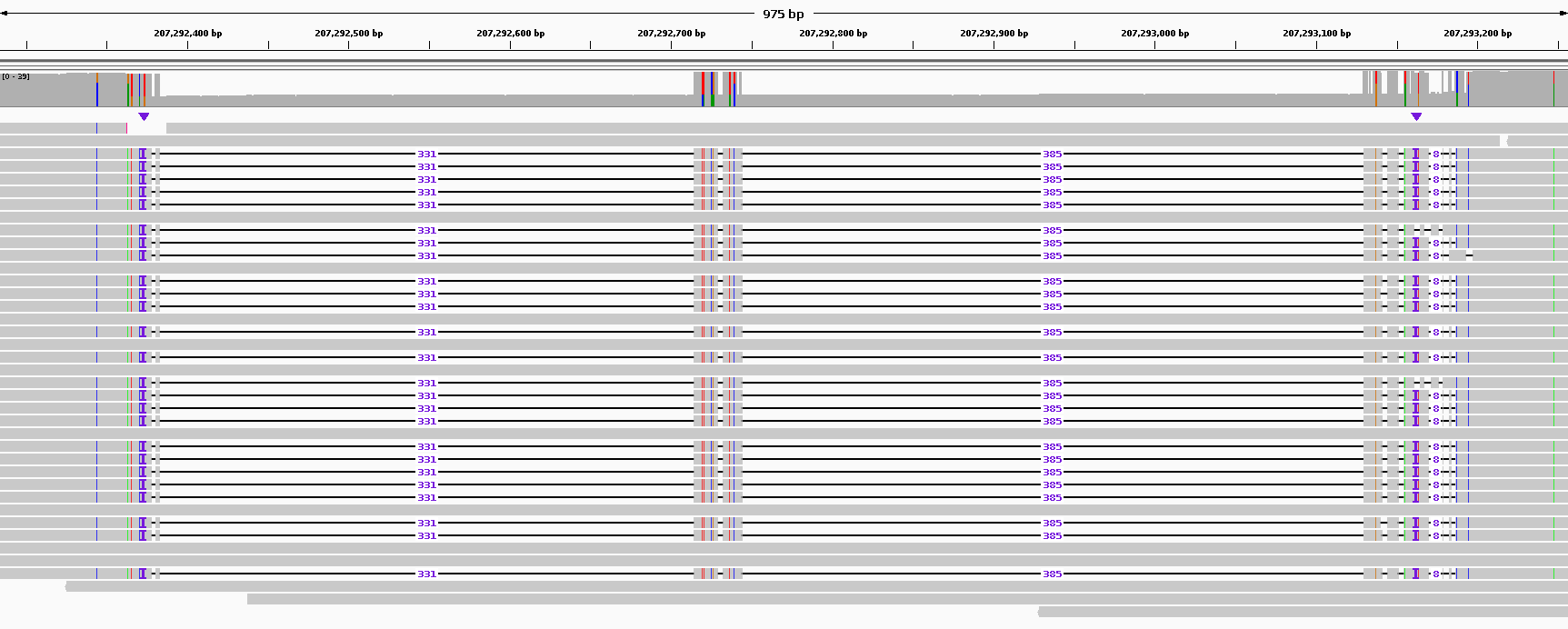

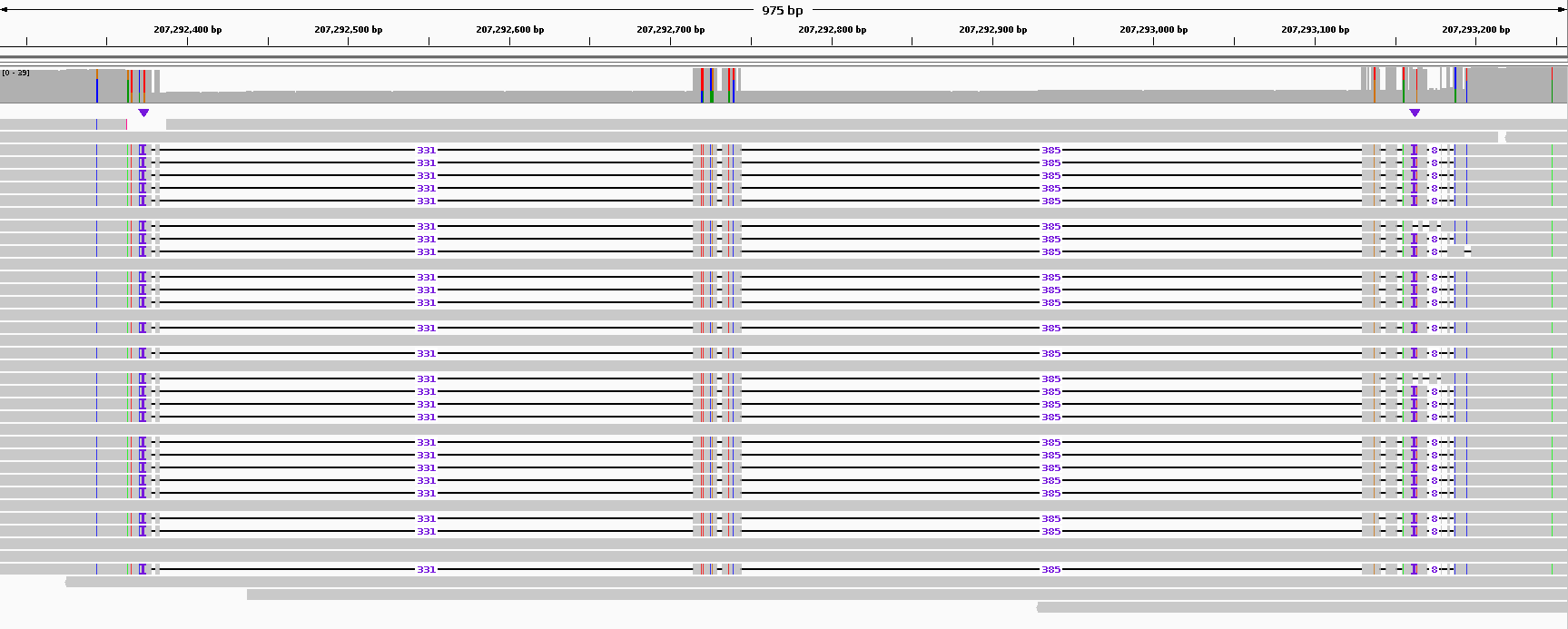


(c) minimap2 (d) Winnowmap2

**Figure S5**. Visualization of alignment pileup near an inversion by using IGV software. This inversion starts at locus 207,292,283 (with length about 800 bp) of chr1. IGV uses purple markers to indicate presence of indels within read alignments. minimap2 and Winnowmap2 show more colour bars due to wrong alignments for this inversion, while invMap and NGMLR shows nearly identical results in this region. IGV can not show the result of lra since error is encountered in querying lra’s alignments.


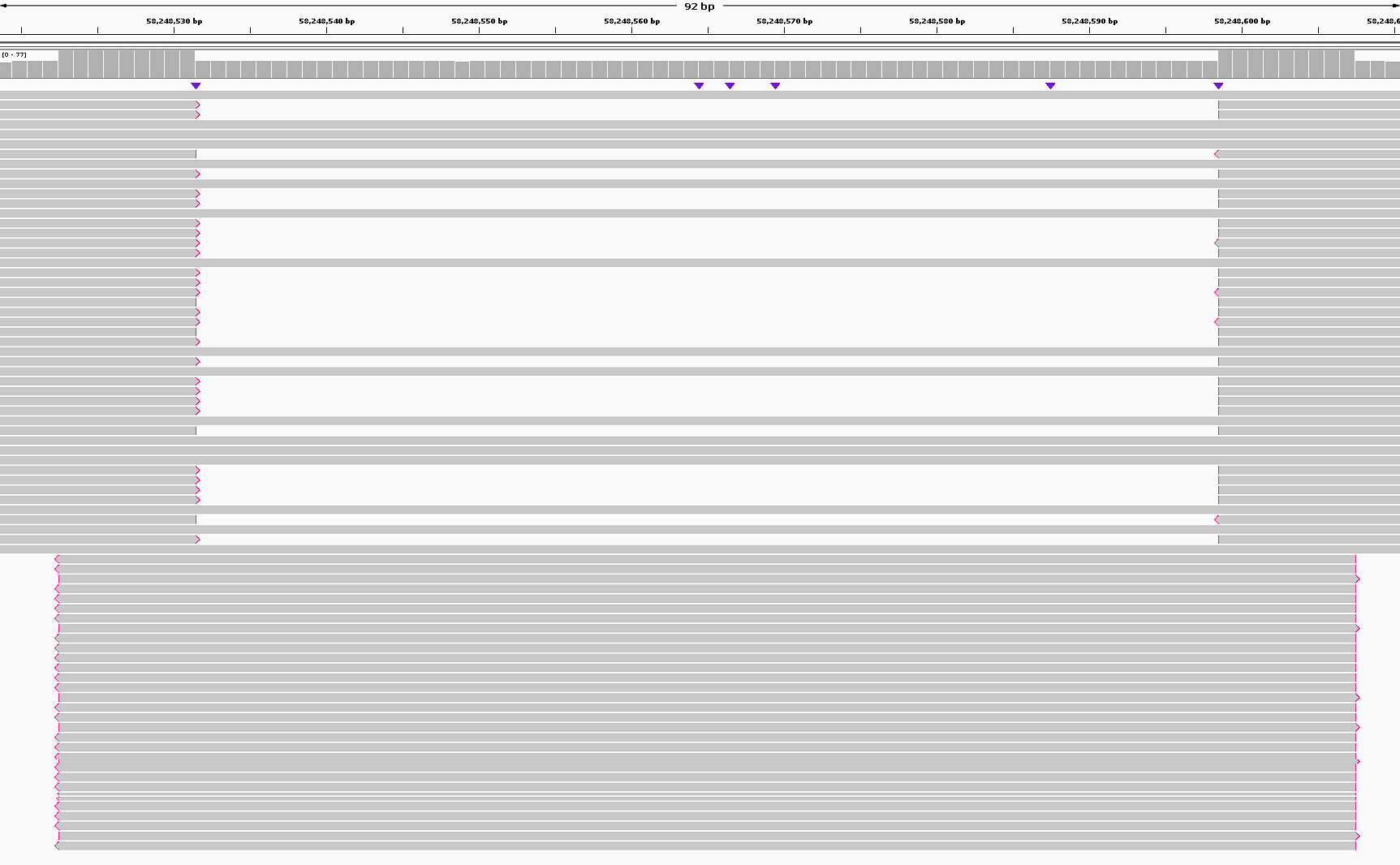

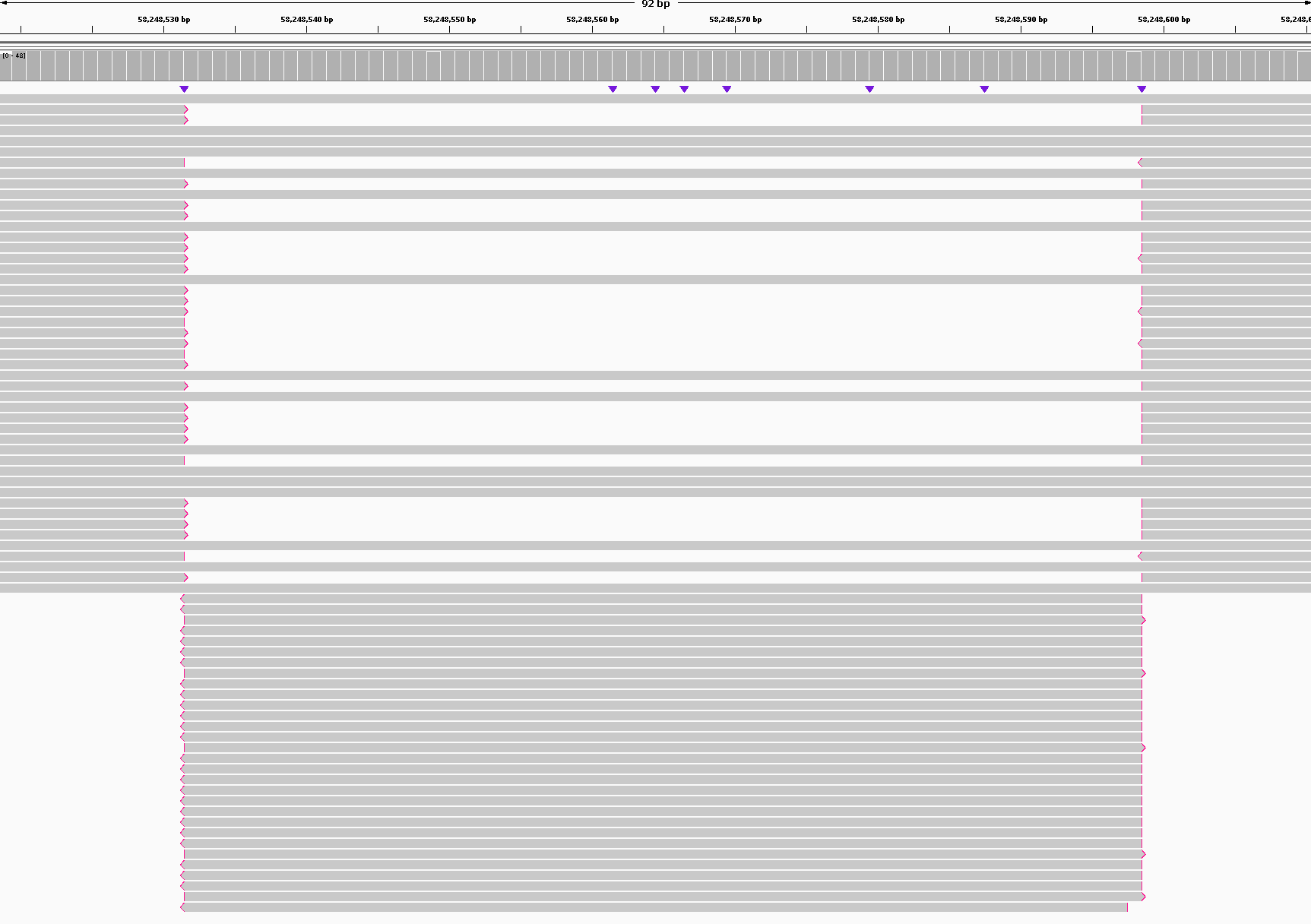


(a) invMap (b) Winnowmap2


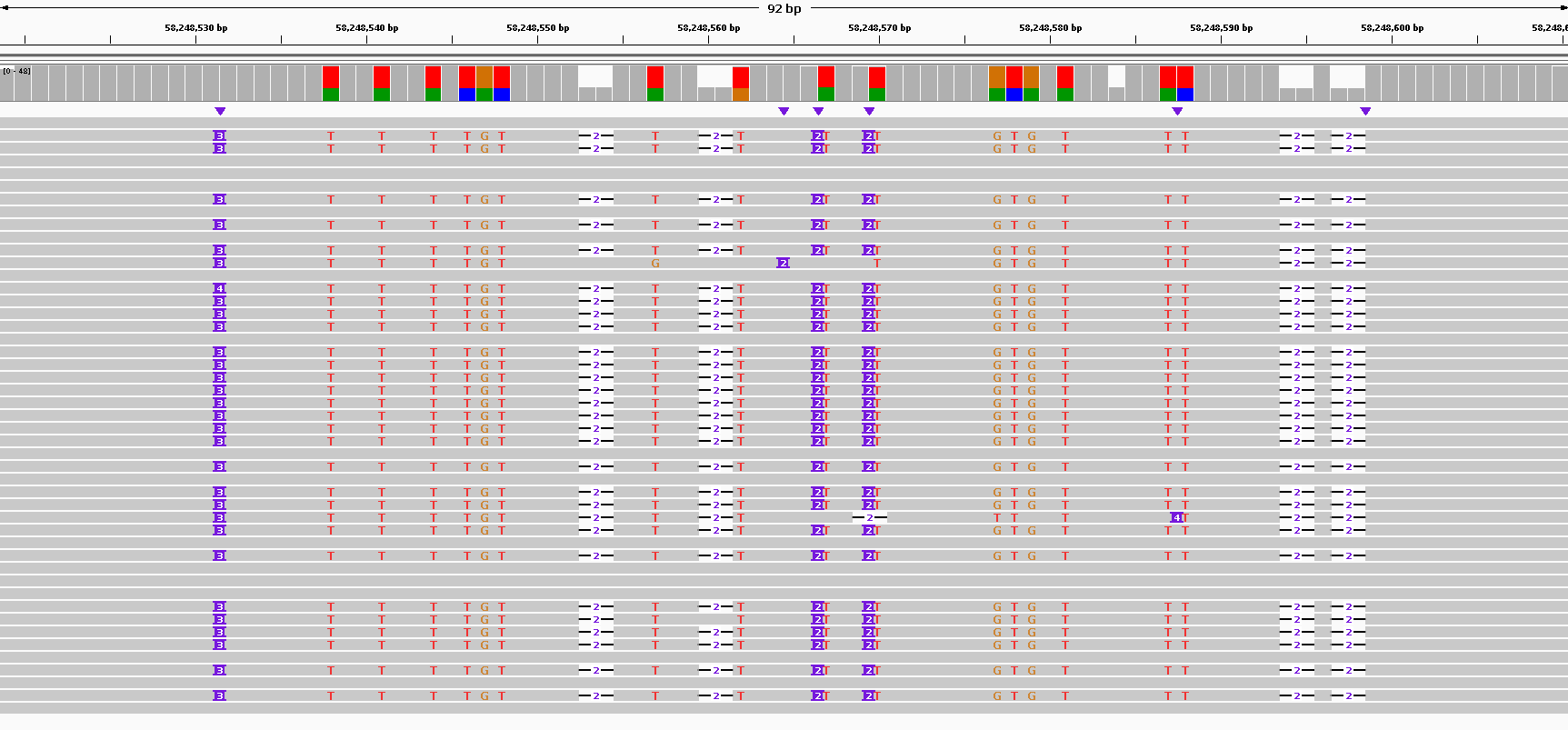

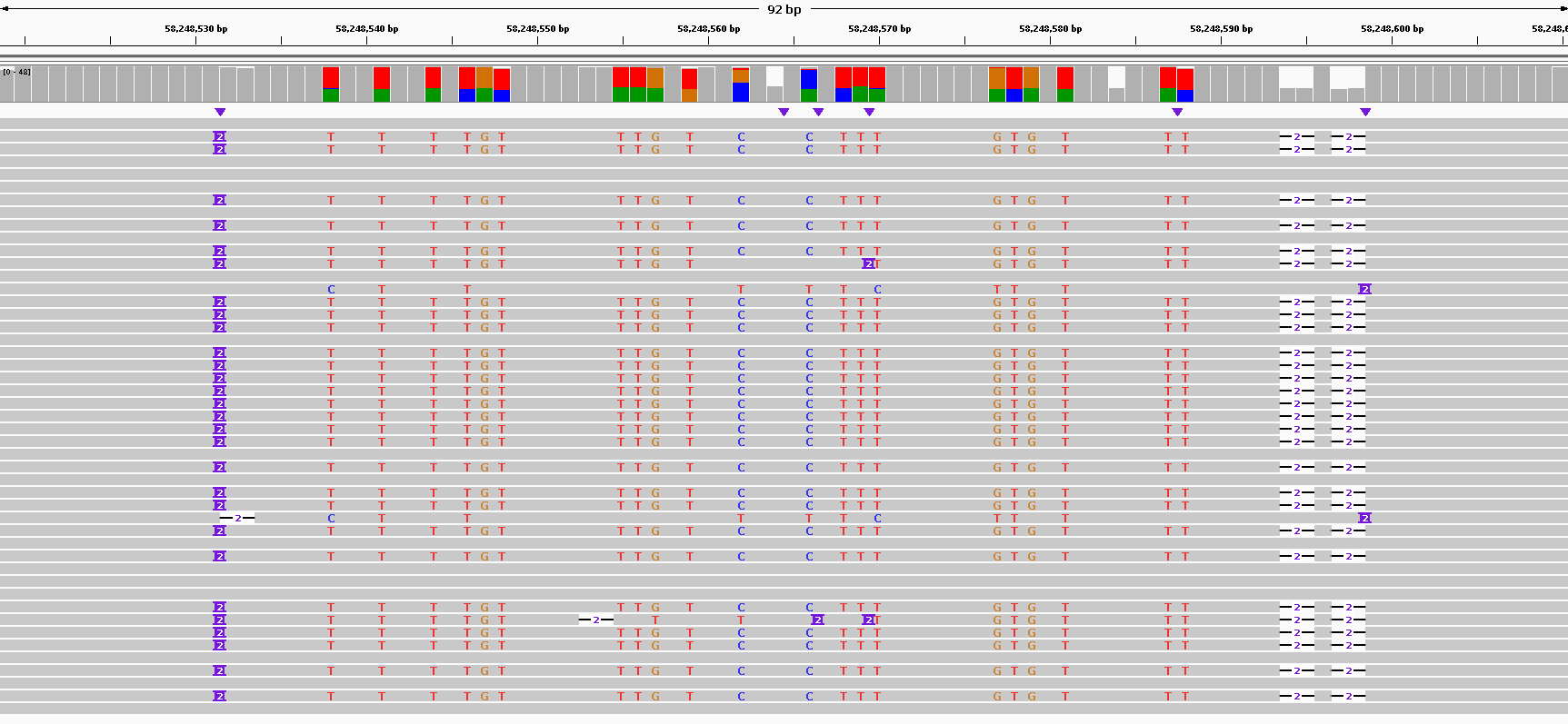


(c) minimap2 (d) NGMLR


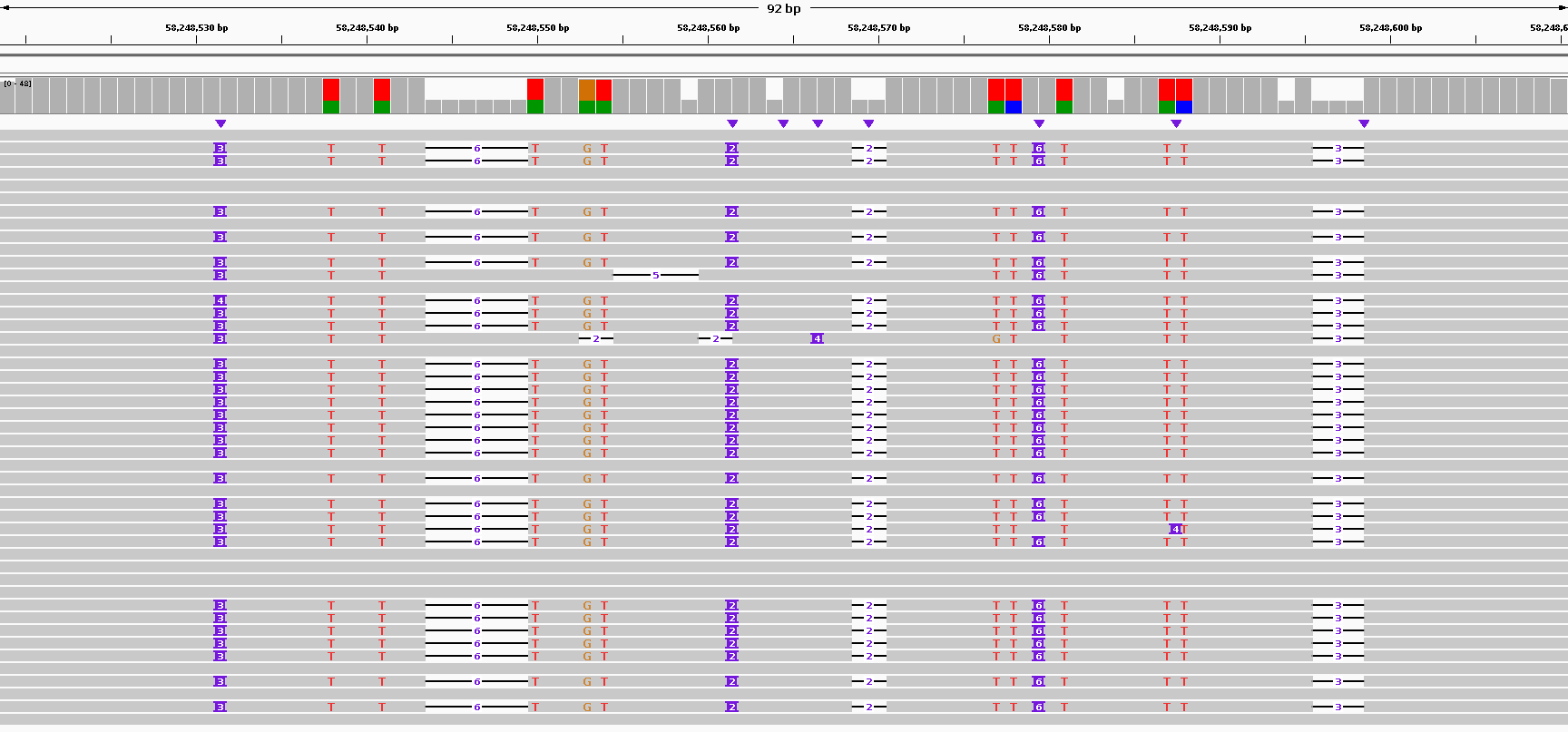


(e) lra

**Figure S6**. Visualization of alignment pileup near an inversion by using IGV software. This inversion starts at locus 58,248,522 (with length about 85 bp) of chr18. IGV uses purple markers to indicate presence of indels within read alignments. minimap2, NGMLR and lra show more colour bars due to wrong alignments for inversion, while invMap and Winnowmap2 show nearly identical results in this region.


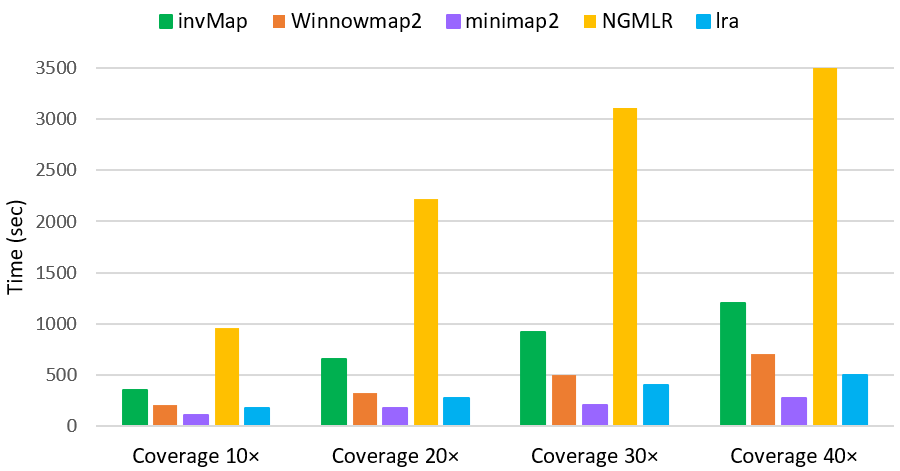


**Figure S7**. Runing time (wall clock time) of each mapper on the simulated datasets used in experiment 3.3 at different coverage levels.
